# Discovery of plasma proteome markers associated with clinical outcome and immunological stress after cardiac surgery

**DOI:** 10.3389/fcvm.2023.1287724

**Published:** 2023-12-22

**Authors:** Corina Bello, Mark G. Filipovic, Markus Huber, Sarah Flannery, Beatrice Kobel, Roman Fischer, Benedikt M. Kessler, Lorenz Räber, Frank Stueber, Markus M. Luedi

**Affiliations:** ^1^ Department of Anaesthesiology and Pain Medicine, Inselspital, Bern University Hospital, University of Bern, Bern, Switzerland; ^2^ Nuffield Department of Medicine, Target Discovery Institute, Centre for Medicines Discovery, University of Oxford, Oxford, United Kingdom; ^3^ Department for BioMedical Research (DBMR), University of Bern, Bern, Switzerland; ^4^ Department of Cardiology, Bern University Hospital, University of Bern, Bern, Switzerland

**Keywords:** cardiopulmonary bypass, acute phase, proteomics, proteome, outcome, cardiovascular surgery, cardiac surgery

## Abstract

**Background:**

Molecular mechanisms underlying perioperative acute phase reactions in cardiac surgery are largely unknown. We aimed to characterise perioperative alterations of the acute phase plasma proteome in a cohort of adult patients undergoing on-pump cardiac surgery using high-throughput mass spectrometry and to identify candidate proteins potentially relevant to postoperative clinical outcome through a novel, multi-step approach.

**Methods:**

This study is an analysis of the Bern Perioperative Biobank, a prospective cohort of adults who underwent cardiac surgery with the use of cardiopulmonary bypass (CPB) at Bern University Hospital between January and December 2019. Blood samples were taken before induction of anaesthesia and on postoperative day one. Proteomic analyses were performed by mass spectrometry. Through a multi-step, exploratory approach, hit-proteins were first identified according to their perioperative prevalence and dynamics. The set of hit-proteins were associated with predefined clinical outcome measures (all-cause one-year mortality, length of hospital stay, postoperative myocardial infarction and stroke until hospital discharge).

**Results:**

192 patients [75.5% male, median age 67.0 (IQR 60.0–73.0)] undergoing cardiac surgery with the use of CPB were included in this analysis. In total, we identified and quantified 402 proteins across all samples, whereof 30/402 (7%) proteins were identified as hit-proteins. Three hit-proteins—LDHB, VCAM1 and IGFBP2—demonstrated the strongest associations with clinical outcomes. After adjustment both for age, sex, BMI and for multiple comparisons, the scaled preoperative levels of IGFBP2 were associated with 1-year all-cause mortality (OR 10.63; 95% CI: 2.93–64.00; *p* = 0.046). Additionally, scaled preoperative levels of LDHB (OR 5.58; 95% CI: 2.58–8.57; *p* = 0.009) and VCAM1 (OR 2.32; 95% CI: 0.88–3.77; *p* = 0.05) were found to be associated with length of hospital stay.

**Conclusions:**

We identified a subset of promising candidate plasma proteins relevant to outcome after on-pump cardiac surgery. IGFBP2 showed a strong association with clinical outcome measures and a significant association of preoperative levels with 1-year all-cause mortality. Other proteins strongly associated with outcome were LDHB and VCAM1, reflecting the dynamics in the acute phase response, inflammation and myocardial injury. We recommend further investigation of these proteins as potential outcome markers after cardiac surgery.

**Clinical Trial Registration:**

ClinicalTrials.gov; NCT04767685, data are available via ProteomeXchange with identifier PXD046496.

## 1. Introduction

Invasive procedures resulting in an altered tissue integrity or contact with foreign materials, such as surgery or the use of cardiopulmonary bypass (CPB), are a source of trauma that induce a variety of inflammatory reactions (1). Such alterations of the immunological homeostasis have a critical impact on patient outcome (1, 2). In cardiac surgery, suspected contributing factors range from effects of the blood contacting the extracorporeal circuit, the sheer stress, the surgical trauma *per se* or lung reperfusion injury upon discontinuation of bypass (3). Nevertheless, despite decades of research, the exact mechanisms behind the systemic inflammatory response, damages of ischemia-reperfusion and subsequent high risk of organ injury and postoperative morbidity in patients undergoing CPB are not well understood (4). Moreover, we are currently unable to reliably identify patients at risk of a pronounced inflammatory response and thus complicated postoperative course.

At least in part, this gap of knowledge can be attributed to the complexity of inflammatory mechanisms, which typically involve a wide array of interrelated pathways, metabolites and interactions. Changes in functional immunity cannot be adequately assessed by routine inflammatory biomarkers such as C-reactive protein, procalcitonin, or numerical analysis of leukocyte (sub)-counts (2). In this context, proteomic analysis includes ideal means to analyse underlying mechanisms of diseases, medical treatments or complex critical conditions such as inflammatory responses to CPB through time-dependent analysis of activated proteins (5). Previous plasma proteomics studies revealed acute phase and inflammatory responses in response to surgical trauma (6), representing a part of complex immunological alterations following surgical stress (7–9), which are highly relevant to clinical outcome: Perioperative hyperinflammation may not only lead to organ dysfunction and death through a massive liberation of pro-inflammatory mediators (10) but can also induce a state of immunosuppression predisposing the individual to post-operative septic complications and infections (11). Identifying those at risk and seeking to attenuate or modulate the response to surgical trauma has the potential to reduce postoperative mortality and morbidity and save billions of dollars in healthcare costs (12).

However, the vast amount of data generated by proteomic analyses presents a challenge to both clinicians and statisticians (13). Tackling this challenge requires innovative methodologic approaches, but might offer the possibility to improve our understanding of underlying mechanisms in perioperative inflammation and may help to identify new prognostic markers and therapeutic targets, which could ultimately improve perioperative care of the individual patient.

Therefore, we aimed to characterise the perioperative acute phase response through mass-spectrometric analysis of the acute phase proteome in a cohort of adult patients undergoing on-pump cardiac surgery. Through application of a novel, multi-step approach including the association with clinical endpoints, we wanted to identify a subset of hit-proteins among the list of proteins detected by mass-spectrometry, which are likely to play a key role in the perioperative acute phase reaction in cardiac surgery patients and which are potentially relevant to postoperative clinical outcome.

## 2. Materials and methods

### 2.1. Study design and study population

This study is an analysis of the Bern Perioperative Biobank (ClinicalTrials.gov; NCT04767685; Submitted: December 16, 2020; principal investigator: Markus M Luedi), a prospective cohort of 192 adult patients undergoing non-emergency cardiac surgery at the Bern University Hospital between January 2019 and December 2019, described elsewhere (14, 15). In brief, all patients underwent elective cardiac surgery (coronary artery bypass grafting, replacement or repair of aortic, mitral or tricuspid valves, surgery of the ascending aorta or aortic arch) with the use of conventional extracorporeal circulation circuits or minimally invasive extracorporeal circulation circuits (16). Emergency surgery or the presence of (suspected) pregnancy were exclusion criteria. Written informed consent was obtained from all participants and the local ethics committee approved the study (Cantonal Ethics Commission of Bern, Bern, CH—KEK Nr. 2018-01272 for sampling and KEK Nr. 2019–2000 for data analysis). Strengthening the Reporting of Observational Studies in Epidemiology (STROBE) guidelines were followed throughout the manuscript.

### 2.2. Blood sampling and acute phase proteome analysis

The mass spectrometry proteomics data have been deposited to the ProteomeXchange Consortium via the PRIDE (17) partner repository with the dataset identifier PXD046496. Blood samples (EDTA) were collected before induction of general anaesthesia (preoperative) and 24 h after surgery (postoperative) and stored at the Bern Liquid Biobank as described before (14, 15). Samples were diluted 10-fold in 5% sodium dodecyl sulphate, 100 mM triethylammonium bicarbonate pH 7.5 then centrifuged at 2,500 rcf for 15 min at ambient temperature. The supernatant was taken and subjected to cysteine reduction and alkylation for 30 min at ambient temperature with 10 mM tris(2-carboxyethyl) phosphine and 50 mM iodoacetamide, respectively. Samples were then processed by S-trap 96-well plate protocol (Protifi) according to the manufacturer's instructions. Digestion was performed overnight at 37°C with 5 µg trypsin (TPCK-treated, Worthington) per 3 *µ*l processed plasma. The resulting peptide samples were dried by vacuum centrifugation then reconstituted in 3% acetonitrile, 0.1% formic acid prior to MS analysis. Peptide samples were loaded onto Evotips (Evosep) according to the manufacturer's instructions and analysed by LC-MS/MS using an Evosep One liquid chromatography system coupled to a TimsTOF Pro mass spectrometer (Bruker). Peptides were chromatographically separated using the 60 samples per day standard Evosep method. The mass spectrometer was operated in diaPASEF mode using 8 diaPASEF scans per TIMS-MS scan. The ion mobility range was set to 0.85–1.3 Vs/cm^2^. Each mass window isolated was 25 m/z wide, ranging from 475 to 1,000 m/z with an ion mobility-dependent collision energy that increased linearly from 20 eV to 59 eV between 0.6–1.6 Vs/cm^2^. Raw MS data were searched in DIA-NN v1.8 against the UniProt human proteome database (UP000000589, downloaded 30th May 2022) plus common contaminants. Identified peptides were permitted a maximum of 1 missed cleavage and cysteine carbamidomethylation was set as a fixed modification, with the MS1 and MS2 mass accuracies both set to 10 ppm, and both match-between-runs and RT-dependent cross-run normalisation enabled.

Conventional CRP was analyzed according to standardized routine laboratory methods (cobas® CRP4 by Roche Diagnostics GmbH, Mannheim, Germany). The perioperative CRP measurements featured values below the detection threshold of 3 mg/L. These values were set to a default value of 2.99 mg/L.

### 2.3. Clinical outcome measures and other study variables

Clinical outcome measures for this analysis were all-cause one-year mortality, length of hospital stay as well as periprocedural myocardial infarction and stroke (recorded until hospital discharge). Myocardial infarction was adjudicated according to the fourth universal definition of myocardial infarction (18) and periprocedural stroke was defined as acute clinically overt neurological deficit with imaging evidence of cerebral ischemia or bleeding.

Pre-, peri-, and postoperative data for each patient were collected from electronic patient charts (Dendrite Clinical Systems Ltd., Henley on-Thames, UK). Information on all-cause mortality was obtained from internal hospital records or from the national records. European System for Cardiac Operative Risk Evaluation (EuroSCORE) II was calculated to assess the presumed risk of 30-day all-cause mortality (19).

### 2.4. Multi-step hit detection procedure

**Figure 1:**
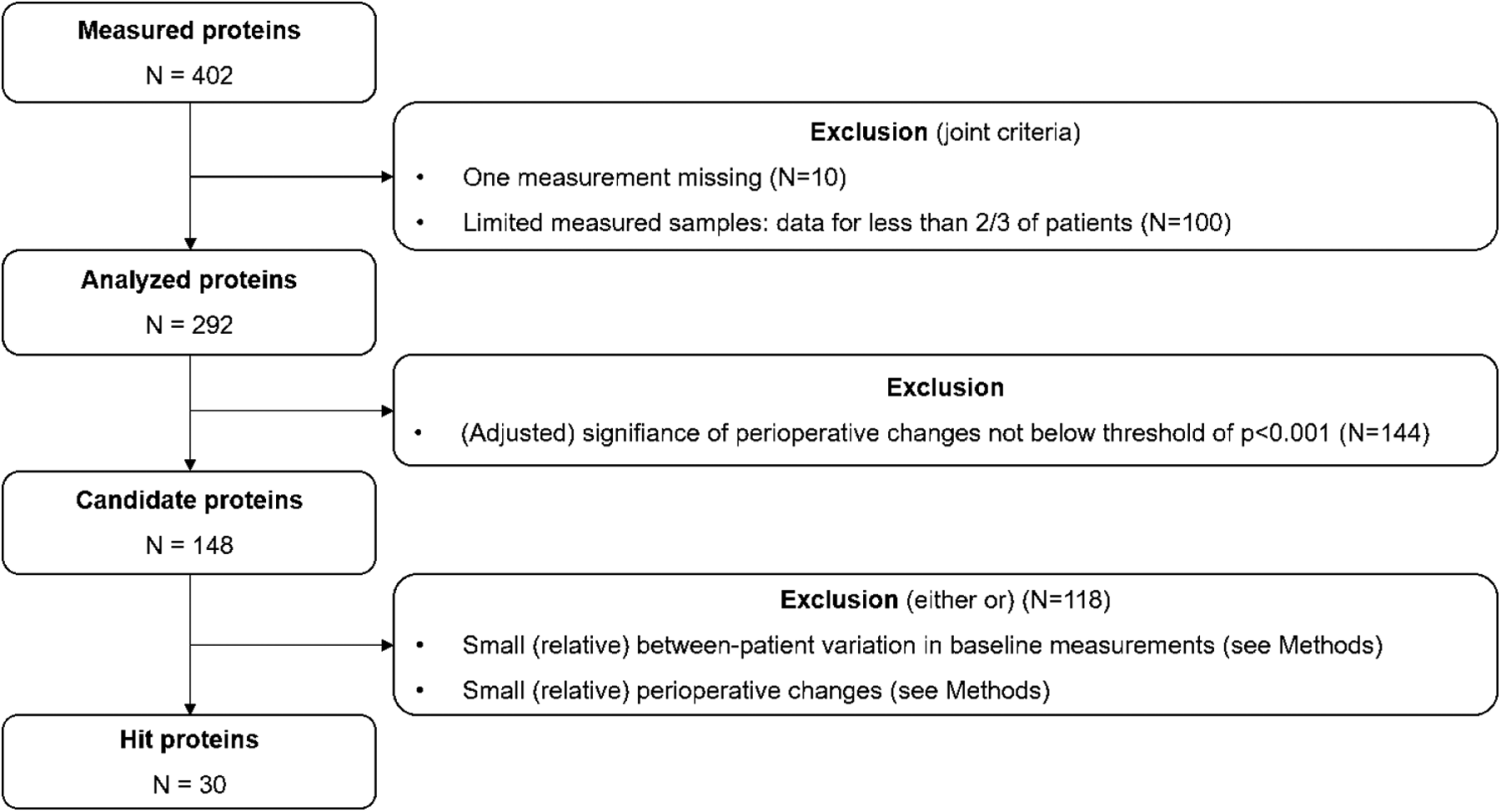
Flow chart of the hit protein selection process.

A graphical representation of our hit protein selection process is provided in Figure 1.

Initially, we imposed two data availability criteria: First, a measurement of a particular protein should be available both pre- and post-operatively. Second, a measurement of a particular protein should be available in at least two-thirds of the patients. We refer to the remaining set of proteins as the *analysed proteins*. Note that proteins that are not present at all or only present in a minority of patients preoperatively, but emerge during or after the surgery, are therefore—by definition—excluded from this analysis.

In addition, the analysed acute phase proteins were grouped into Kyoto Encyclopedia of Genes and Genomes (KEGG) PATHWAYS according to the KEGG-PATHWAY database (20), using the following KEGG-PATHWAY-classifications: metabolism, genetic information processing, environmental information processing, cellular processes, organismal systems, multiple categories, unknown/unclassified. This step served as a purely exploratory measure to guide the interpretation of the hit selection procedure.

To be able to compare different proteins, we scaled each protein measurement by first subtracting the median value (pooled across the two time points) and then by dividing the residual by the interquartile range; the scaling is thus similar to the computation of a *z*-score, but accounts for possible skewness in the measured values by considering the median and interquartile range instead of the mean and standard deviation.

As a next step, we assessed the statistical significance of the perioperative change in the analysed proteins by means of a paired samples Wilcoxon test. Given the large number of candidate proteins, the resulting *p*-values were adjusted for multiple comparison by means of the Bonferroni correction. Only proteins with adjusted *p*-values < 0.001 were subsequently considered: we denote this set as the *candidate proteins*.

As final step, a two-dimensional, empirical approach was chosen to determine the final set of hit proteins. The first dimension considers the perioperative change in protein levels among the candidate proteins, which should show either a large decrease or large increase perioperatively. The second dimension requires hit proteins to demonstrate some degree of inter-patient variability: if there is no inter-patient variability, for example all patients feature exactly the same (statistically significant) increase in protein levels, then this change is unlikely to be related to a particular outcome such as length of hospital stay (which does feature inter-patient variability). The joint consideration of these two dimensions defines an area of interest in a two-dimensional space defined by the (scaled) median perioperative change in protein levels (***x***) and the variation (defined by means of the interquartile range) in perioperative protein levels (***y***).

We then defined a distance (***d***) in this space as follows: ***d***^2^ = 0.66*x^2^ + 0.33*y^2^, thus defining an ellipse in the two-dimensional space. The final set of hit proteins is defined as those 20% of proteins with the largest distances: for these proteins, the distance is greater than the 80%-quantile of all distances [denoted as ***d*_q(0.8)_**]. All computations were performed with R version 4.0.2 (21).

### 2.5. Outcome analysis

For the final set of hit proteins, we computed both crude and adjusted (age, gender and BMI) odds ratios (ORs) with the four clinical outcomes of interest by means of univariable and multivariable logistic regression. For each clinical outcome individually, the *p*-values resulting from this analysis were adjusted for multiple comparison by means of the Bonferroni correction. For exploratory purposes, we further calculated univariable association of preoperative protein levels as well as their perioperative changes with a set of baseline covariates (Supplementary Material).

## 3. Results

### 3.1. Patient / study population characteristics

A total of 192 patients [75.5% male, median age 67.0 (IQR 60.0–73.0)] undergoing cardiac surgery with the use of CPB were included in this analysis. Both baseline characteristics as well as procedural and surgical characteristics have been extensively described previously (Table 1) (14, 15). Mean CRP levels measured with conventional routine laboratory methods at baseline were 3.0 mg/dl [3.0; 3.2] and fully reported in Supplementary Table S2 for the perioperative period.

**Table 1:** Patient and surgical characteristics. Both baseline characteristics as well as procedural and surgical characteristics have been extensively described previously (14, 15).

	All patients	*N*
	*N = 192*	
Age (years)	67 [60;73]	192
Sex (female)	47 (24.5%)	192
BMI (kg/m^2^)	26.1 [23.7;30.4]	192
Diabetes on insulin (Yes)	35 (18.2%)	192
Hypertension (Yes)	130 (68.4%)	190
Dyslipidemia (Yes)	111 (58.1%)	191
Smoker:		188
Non-smoker	97 (51.6%)	
Previous / current smoker	91 (48.4%)	
Obesity (Yes)	52 (27.1%)	192
Preoperative renal disease (Yes)	43 (22.4%)	192
Peripheral vascular disease (Yes)	11 (6.2%)	178
Carotid disease (Yes)	6 (3.7%)	162
Myocardial infarction (Yes)	20 (10.5%)	191
COPD (Yes)	23 (12.1%)	190
NYHA (>1)	131 (68.6%)	191
CCS (>0)	71 (37.6%)	189
Ejection Fraction (%)	60 [55;65]	191
EuroSCORE 2	1.73 [0.90;2.93]	184
ECC or MiECC:		192
ECC	149 (77.6%)	
MiECC	43 (22.4%)	
Deep hypothermic cardiac arrest (Yes)	19 (10.0%)	191
Aortic valve (Yes)	86 (44.8%)	192
Mitral valve (Yes)	45 (23.4%)	192
Tricuspid valve (Yes)	17 (8.9%)	192
Coronary artery bypass (Yes)	77 (40.1%)	192
Ascending Aortic (Yes)	38 (19.8%)	192
Aortic Arch (Yes)	11 (5.7%)	192
Bypass time (min)	104 [80;132]	192
Aortic cross clamping (min)	68.5 [52.0;91.8]	192
Lowest body temperature (°C)	33.2 [32.1;33.8]	192
Operation duration (min)	234 [195;276]	192

Data expressed as median [IQR] or number (%).

BMI, body mass index; COPD, chronic obstructive pulmonary disease; NYHA, New York heart association; CCS, Canadian cardiovascular society.

### 3.2. Multi-step hit detection

**Figure 2:**
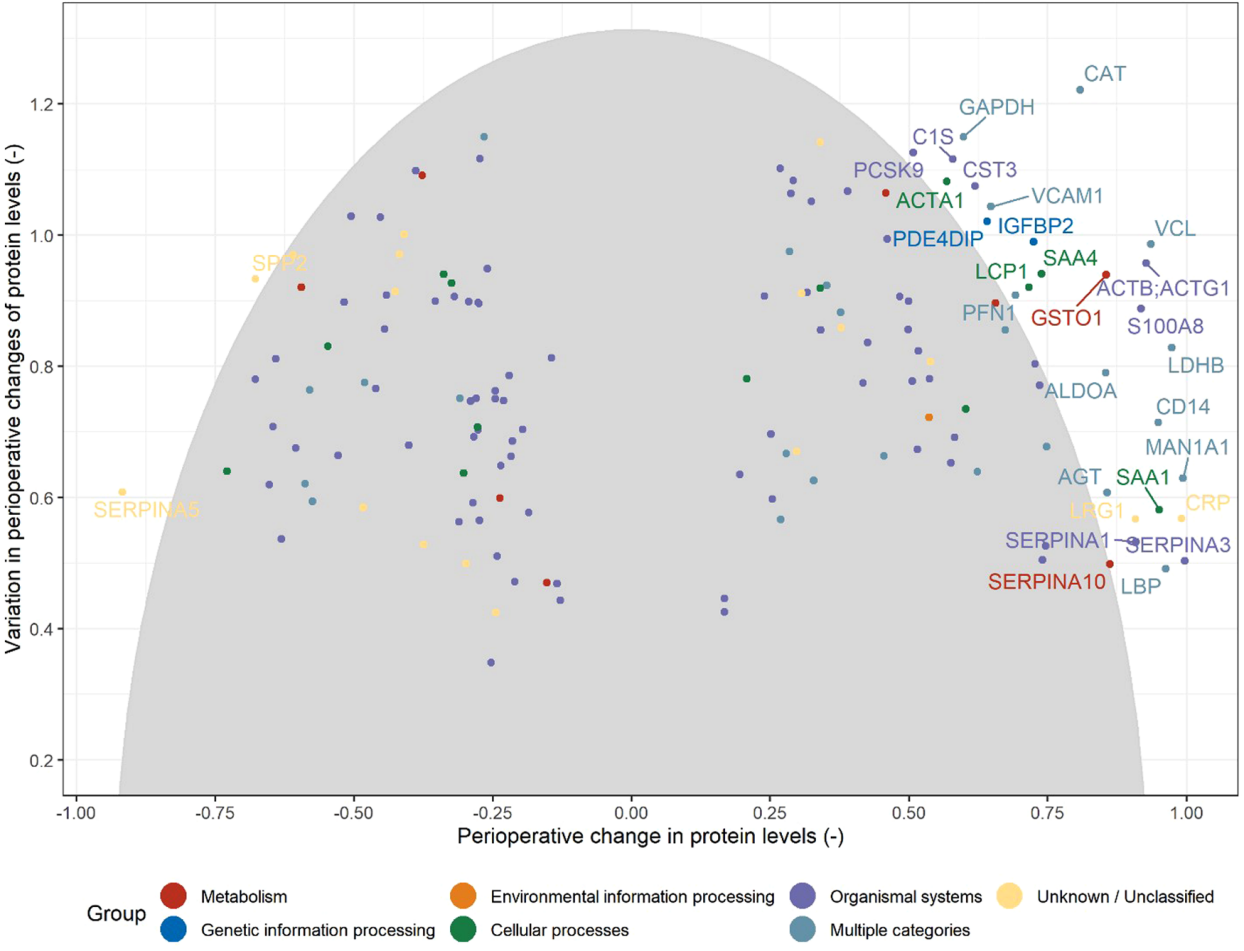
Illustration of the selection of hit proteins from the set of candidate proteins (see Figure 1) based on the magnitude of the change from baseline (preoperative) values and the between-patient variation in the perioperative changes. Note that centered (by the median) and scaled (by the interquartile range) protein levels are used. The grey ellipse includes the 80% of proteins with the shortest distances in this two-dimensional space [e.g. distances below the 80% quantile of all distances: ***d*_q(0.8)_**; see Methods]. Proteins are declared as hits when their corresponding values are above the threshold values [***d*_q(0.8)_**].

In total, we identified 402 proteins in our samples (a list of all detected proteins can be found in the Supplement: Supplementary Table S1). Out of the 402 identified proteins, 292 / 402 (73%) proteins were detectable at both time points of the serum sampling. Of those, 148 / 402 (37%) showed significant perioperative changes at a threshold below *p* < 0.001. After exclusion of all proteins with only small (relative) in-between patient variation regarding both, baseline measurements and perioperative changes, 30 / 402 proteins (7%) were identified as hit-proteins (Figure 2). An overview of the 30 identified hit-proteins including pre- and postoperative levels, perioperative change and KEGG pathway database category (20) is provided in Table 2. Association of unadjusted preoperative protein levels with baseline characteristics is shown in Supplementary Figures S1, S2, and for perioperative change in Supplementary Figures S3, S4, respectively.

**Table 2:** Characteristics of the 30 proteins selected for further analysis. Note that centered (by the median) and scaled (by the interquartile range) protein levels are used in the computations (see Methods).

Protein	Preoperative levels	Postoperative levels	Perioperative change	*p* value	Protein name	Protein description	KEGG pathway database classification	Specification of organismal systems
	Median [IQR]	Median [IQR]	Median [IQR]	(adjusted *p*)				
ACTA1	−0.29 [−0.57 to 0.07]	0.47 [−0.03 to 0.89]	0.57 [0.10 to 1.18]	<0.001 (<0.001)	ACTS_HUMAN	Actin, alpha skeletal muscle	4	
ACTB;ACTG1	−0.42 [−0.69 to −0.10]	0.50 [0.12 to 1.04]	0.93 [0.48 to 1.44]	<0.001 (<0.001)	ACTB_HUMAN; ACTG_HUMAN	Actin, cytoplasmic 1	5	Immune system, endocrine system, digestive system, environmental adaptation
AGT	−0.44 [−0.68 to −0.15]	0.45 [0.10 to 0.78]	0.86 [0.57 to 1.18]	<0.001 (<0.001)	ANGT_HUMAN	Angiotensinogen	3; 5	Endocrine, circulatory
ALDOA	−0.45 [−0.67 to −0.13]	0.44 [0.09 to 0.85]	0.85 [0.51 to 1.30]	<0.001 (<0.001)	ALDOA_HUMAN	Fructose-bisphosphate aldolase A	1; 3	
C1S	−0.24 [−0.65 to 0.16]	0.28 [−0.19 to 0.74]	0.58 [−0.03 to 1.09]	<0.001 (<0.001)	C1S_HUMAN	Complement C1s subcomponent	5	Immune
CAT	−0.43 [−0.70 to −0.01]	0.34 [0.00 to 0.92]	0.81 [0.19 to 1.41]	<0.001 (<0.001)	CATA_HUMAN	Catalase	1; 3; 4; 5	Aging
CD14	−0.41 [−0.64 to −0.15]	0.57 [0.18 to 1.01]	0.95 [0.60 to 1.31]	<0.001 (<0.001)	CD14_HUMAN	Monocyte differentiation antigen CD14	3; 4; 5	Immune
CRP	−0.39 [−0.40 to −0.36]	0.61 [0.36 to 0.92]	0.99 [0.71 to 1.28]	<0.001 (<0.001)	CRP_HUMAN	C-reactive protein	no KO	
CST3	−0.23 [−0.70 to 0.19]	0.22 [−0.18 to 0.92]	0.62 [0.10 to 1.18]	<0.001 (<0.001)	CYTC_HUMAN	Cystatin-C	5	Digestive
GAPDH	−0.28 [−0.71 to 0.27]	0.25 [−0.19 to 0.70]	0.60 [−0.09 to 1.06]	<0.001 (<0.001)	G3P_HUMAN	Glyceraldehyde-3-phosphate dehydrogenase	1; 3	
GSTO1	−0.31 [−0.61 to 0.00]	0.54 [0.00 to 1.17]	0.86 [0.35 to 1.29]	<0.001 (<0.001)	GSTO1_HUMAN	Glutathione S-transferase omega-1	1	
IGFBP2	−0.29 [−0.49 to 0.00]	0.50 [0.00 to 1.08]	0.72 [0.38 to 1.37]	<0.001 (<0.001)	IBP2_HUMAN	Insulin-like growth factor-binding protein 2	2	
LBP	−0.35 [−0.43 to −0.27]	0.65 [0.41 to 0.93]	0.96 [0.75 to 1.24]	<0.001 (<0.001)	LBP_HUMAN	Lipopolysaccharide-binding protein	3; 5	Immune
LCP1	−0.40 [−0.75 to 0.00]	0.43 [0.00 to 0.73]	0.72 [0.30 to 1.22]	<0.001 (<0.001)	PLSL_HUMAN	Plastin-2	4	
LDHB	−0.33 [−0.49 to −0.15]	0.66 [0.20 to 1.21]	0.97 [0.60 to 1.43]	<0.001 (<0.001)	LDHB_HUMAN	L-lactate dehydrogenase B chain	1; 3; 5	Endocrine
LRG1	−0.43 [−0.54 to −0.29]	0.52 [0.25 to 0.90]	0.91 [0.66 to 1.23]	<0.001 (<0.001)	A2GL_HUMAN	Leucine-rich alpha-2-glycoprotein	no KO	
MAN1A1	−0.42 [−0.64 to −0.24]	0.56 [0.27 to 0.90]	0.99 [0.68 to 1.31]	<0.001 (<0.001)	MA1A1_HUMAN	Mannosyl-oligosaccharide 1,2-alpha-mannosidase IA	1; 2	
PCSK9	−0.25 [−0.63 to 0.23]	0.28 [−0.16 to 0.83]	0.51 [−0.09 to 1.03]	<0.001 (<0.001)	PCSK9_HUMAN	Proprotein convertase subtilisin/kexin type 9	5	Digestive
PDE4DIP	−0.33 [−0.53 to 0.04]	0.41 [−0.03 to 0.91]	0.64 [0.16 to 1.18]	<0.001 (<0.001)	MYOME_HUMAN	Myomegalin	2	
PFN1	−0.40 [−0.67 to 0.07]	0.31 [−0.09 to 0.79]	0.69 [0.28 to 1.19]	<0.001 (<0.001)	PROF1_HUMAN	Profilin-1	3; 4	
S100A8	−0.39 [−0.58 to −0.14]	0.55 [0.17 to 1.16]	0.92 [0.56 to 1.45]	<0.001 (<0.001)	S10A8_HUMAN	Protein S100-A8	5	Immune
SAA1	−0.27 [−0.27 to −0.25]	0.73 [0.40 to 0.96]	0.95 [0.62 to 1.20]	<0.001 (<0.001)	SAA1_HUMAN	Serum amyloid A-1 protein	4	
SAA4	−0.33 [−0.61 to 0.03]	0.43 [−0.06 to 0.85]	0.74 [0.27 to 1.21]	<0.001 (<0.001)	SAA4_HUMAN	Serum amyloid A-4 protein	4	
SERPINA1	−0.47 [−0.64 to −0.24]	0.52 [0.26 to 0.74]	0.91 [0.65 to 1.18]	<0.001 (<0.001)	A1AT_HUMAN	Alpha-1-antitrypsin	5	Immune
SERPINA10	−0.47 [−0.66 to −0.23]	0.52 [0.22 to 0.77]	0.86 [0.68 to 1.18]	<0.001 (<0.001)	ZPI_HUMAN	Protein Z-dependent protease inhibitor	1	
SERPINA3	−0.51 [−0.60 to −0.37]	0.49 [0.29 to 0.74]	1.00 [0.74 to 1.25]	<0.001 (<0.001)	AACT_HUMAN	Alpha-1-antichymotrypsin	5	Immune (complement and coagulation)
SERPINA5	0.58 [0.22 to 0.92]	−0.35 [−0.59 to −0.11]	−0.92 [−1.20 to −0.60]	<0.001 (<0.001)	IPSP_HUMAN	Plasma serine protease inhibitor	5	Immune (complement and coagulation)
SPP2	0.47 [−0.07 to 0.96]	−0.26 [−0.54 to 0.06]	−0.68 [−1.16 to −0.23]	<0.001 (<0.001)	SPP24_HUMAN	Secreted phosphoprotein 24	no KO	
VCAM1	−0.24 [−0.69 to 0.10]	0.38 [−0.15 to 0.99]	0.65 [0.19 to 1.24]	<0.001 (<0.001)	VCAM1_HUMAN	Vascular cell adhesion protein 1	3; 5	Immune
VCL	−0.37 [−0.64 to −0.02]	0.51 [0.02 to 1.11]	0.94 [0.47 to 1.46]	<0.001 (<0.001)	VINC_HUMAN	Vinculin	4; 5	Immune

### 3.3. Association of hit-proteins with clinical outcome measures

After adjusting for age, BMI and gender, several hit-proteins baseline spectral counts (*n* = 9/30, 30%) and perioperative changes (*n* = 8/30, 27%) were significantly associated with clinical outcomes (Tables 3, 4). The three hit-proteins which showed the strongest associations with clinical outcomes were VCAM1, LDHB and IGFBP2, while the SERPINs yielded a large number of associations with outcome measures. After adjustment for multiple comparison, the associations of preoperative levels of IGFBP2 with 1-year all-cause mortality (OR 10.63; 95% CI: 2.93–64.00; p = 0.046), and the associations of preoperative levels of LDHB (OR 5.58; 95% CI: 2.58–8.57; *p* = 0.009) and VCAM1 (OR 2.32; 95% CI: 0.88–3.77; *p* = 0.05) with length of hospital stay remained significant (Table 3), while no association of perioperative change of any protein with clinical outcome measures could be observed (Table 4).

**Table 3:** Adjusted associations of preoperative protein levels with postoperative outcome. Odds ratios (ORs) and 95%-confidence intervals derived from a multivariable linear regression are shown for binary outcomes, whereas regression coefficient and their 95%-confidence intervals computed with a multivariable linear regression are shown for the continuous outcome length of hospital stay. The associations are adjusted for age, gender and BMI. *P*-values are adjusted for multiple comparison for each outcome separately by means of the Bonferroni correction.

Protein	Stroke		Myocardial infarction		1-year mortality		Length of stay	
	Incidence 12/192 (6.3%)		Incidence 6/192 (3.1%)		Incidence 5/192 (2.6%)		Median 7 [IQR: 6–9 ] days	
	OR (95%-CI)	*p* (adjusted *p*)	OR (95%-CI)	*p* (adjusted *p*)	OR (95%-CI)	*p* (adjusted *p*)	Beta (95%-CI)	*p* (adjusted *p*)
ACTA1	1.07 (0.24–3.88)	0.92 (>0.99)	0.04 (0.00–1.00)	0.13 (>0.99)	1.56 (0.23–7.67)	0.60 (>0.99)	0.54 (−1.96–3.05)	0.67 (>0.99)
ACTB;ACTG1	0.98 (0.18–4.48)	0.98 (>0.99)	0.10 (0.00–1.24)	0.13 (>0.99)	1.26 (0.13–8.62)	0.82 (>0.99)	1.40 (−0.67–3.46)	0.18 (>0.99)
AGT	1.18 (0.35–3.10)	0.75 (>0.99)	0.05 (0.00–0.67)	0.039 (>0.99)	0.85 (0.08–3.31)	0.86 (>0.99)	2.23 (0.35–4.10)	0.02 (0.61)
ALDOA	1.44 (0.30–5.26)	0.61 (>0.99)	0.21 (0.02–1.50)	0.18 (>0.99)	0.64 (0.04–4.16)	0.70 (>0.99)	1.47 (−0.60–3.53)	0.16 (>0.99)
C1S	0.59 (0.24–1.52)	0.27 (>0.99)	1.84 (0.35–11.78)	0.49 (>0.99)	0.78 (0.24–2.95)	0.70 (>0.99)	0.31 (−0.93–1.56)	0.62 (>0.99)
CAT	1.08 (0.37–2.38)	0.86 (>0.99)	0.03 (0.00–0.70)	0.07 (>0.99)	0.46 (0.03–2.21)	0.49 (>0.99)	0.58 (−0.85–2.00)	0.43 (>0.99)
CD14	1.51 (0.24–9.15)	0.65 (>0.99)	1.19 (0.12–9.17)	0.87 (>0.99)	2.49 (0.20–26.48)	0.46 (>0.99)	−0.90 (−3.43–1.63)	0.48 (>0.99)
CRP	0.35 (0.00–10.49)	0.70 (>0.99)	0.00 (0.00–6.39)	0.29 (>0.99)	2.03 (0.00–40.35)	0.70 (>0.99)	1.27 (−4.84–7.39)	0.68 (>0.99)
CST3	1.56 (0.85–2.73)	0.12 (>0.99)	0.13 (0.01–0.73)	0.049 (>0.99)	2.08 (0.90–4.49)	0.06 (>0.99)	1.02 (−0.09–2.14)	0.07 (>0.99)
GAPDH	0.47 (0.15–1.24)	0.17 (>0.99)	0.47 (0.10–1.57)	0.29 (>0.99)	0.91 (0.22–1.81)	0.87 (>0.99)	−0.45 (−1.31–0.41)	0.30 (>0.99)
GSTO1	0.80 (0.14–3.34)	0.79 (>0.99)	0.07 (0.00–1.63)	0.20 (>0.99)	0.63 (0.03–4.52)	0.72 (>0.99)	0.81 (−1.15–2.78)	0.41 (>0.99)
IGFBP2	1.12 (0.20–3.90)	0.88 (>0.99)	0.24 (0.00–2.73)	0.46 (>0.99)	10.63 (2.93–64.00)	0.002 (0.046)	2.17 (0.25–4.09)	0.027 (0.82)
LBP	2.57 (0.03–67.82)	0.62 (>0.99)	0.50 (0.00–102.71)	0.82 (>0.99)	1.09 (0.00–68.90)	0.97 (>0.99)	1.86 (−3.63–7.35)	0.50 (>0.99)
LCP1	2.08 (0.81–4.80)	0.09 (>0.99)	1.25 (0.20–5.11)	0.78 (>0.99)	0.64 (0.10–2.44)	0.59 (>0.99)	1.48 (0.10–2.86)	0.035 (>0.99)
LDHB	1.60 (0.22–10.29)	0.63 (>0.99)	0.65 (0.02–13.32)	0.80 (>0.99)	0.61 (0.02–10.71)	0.77 (>0.99)	5.58 (2.58–8.57)	0.0003 (0.009)
LRG1	1.09 (0.07–6.77)	0.94 (>0.99)	0.08 (0.00–2.53)	0.34 (>0.99)	4.89 (0.59–28.23)	0.09 (>0.99)	1.29 (−1.77–4.34)	0.41 (>0.99)
MAN1A1	0.28 (0.02–2.41)	0.32 (>0.99)	2.55 (0.09–36.24)	0.55 (>0.99)	0.55 (0.02–5.54)	0.70 (>0.99)	−0.93 (−3.37–1.50)	0.45 (>0.99)
PCSK9	1.25 (0.33–2.78)	0.66 (>0.99)	1.66 (0.54–3.52)	0.24 (>0.99)	0.93 (0.15–2.40)	0.92 (>0.99)	−0.02 (−1.19–1.16)	0.98 (>0.99)
PDE4DIP	0.33 (0.02–1.08)	0.26 (>0.99)	1.29 (0.38–3.15)	0.61 (>0.99)	0.88 (0.19–1.92)	0.81 (>0.99)	0.08 (−1.02–1.19)	0.88 (>0.99)
PFN1	0.49 (0.08–2.16)	0.39 (>0.99)	0.11 (0.00–1.00)	0.15 (>0.99)	1.29 (0.18–7.77)	0.79 (>0.99)	0.50 (−1.53–2.53)	0.63 (>0.99)
S100A8	0.16 (0.01–1.27)	0.17 (>0.99)	0.67 (0.04–2.26)	0.61 (>0.99)	1.25 (0.25–2.87)	0.67 (>0.99)	−0.02 (−1.59–1.56)	0.98 (>0.99)
SAA1	0.07 (0.00–4.93)	0.68 (>0.99)	0.39 (0.00–37.64)	0.81 (>0.99)	1.05 (NA^a^ - 10.50)	0.98 (>0.99)	−0.17 (−4.25–3.91)	0.93 (>0.99)
SAA4	0.62 (0.16–1.98)	0.44 (>0.99)	0.45 (0.05–2.38)	0.40 (>0.99)	0.75 (0.11–3.81)	0.75 (>0.99)	0.22 (−1.50–1.94)	0.80 (>0.99)
SERPINA1	0.53 (0.06–3.67)	0.54 (>0.99)	0.01 (0.00–0.78)	0.08 (>0.99)	5.13 (0.51–43.46)	0.14 (>0.99)	3.75 (1.27–6.24)	0.003 (0.1)
SERPINA10	0.67 (0.10–3.79)	0.66 (>0.99)	1.95 (0.09–43.22)	0.66 (>0.99)	1.89 (0.13–22.55)	0.62 (>0.99)	−0.26 (−2.98–2.47)	0.85 (>0.99)
SERPINA3	1.29 (0.06–11.40)	0.85 (>0.99)	0.37 (0.00–5.93)	0.56 (>0.99)	7.76 (0.80–61.44)	0.044 (>0.99)	1.25 (−2.17–4.68)	0.47 (>0.99)
SERPINA5	0.29 (0.09–0.92)	0.038 (>0.99)	1.52 (0.30–9.51)	0.63 (>0.99)	0.24 (0.04–1.17)	0.08 (>0.99)	0.22 (−1.39–1.83)	0.79 (>0.99)
SPP2	0.71 (0.31–1.49)	0.38 (>0.99)	1.18 (0.30–4.40)	0.81 (>0.99)	1.12 (0.34–3.43)	0.85 (>0.99)	−0.84 (−2.08–0.40)	0.18 (>0.99)
VCAM1	1.80 (0.80–4.01)	0.15 (>0.99)	0.50 (0.05–2.94)	0.50 (>0.99)	1.92 (0.54–5.73)	0.26 (>0.99)	2.32 (0.88–3.77)	0.002 (0.05)
VCL	1.60 (0.26–9.58)	0.60 (>0.99)	0.09 (0.00–3.23)	0.26 (>0.99)	1.58 (0.18–12.07)	0.66 (>0.99)	1.98 (−0.59–4.54)	0.13 (>0.99)

^a^ Lower bound of profile likelihood confidence interval could not be computed.

**Table 4:** Adjusted associations of perioperative change in proteins levels with postoperative outcome. Odds ratios (ORs) and 95%-confidence intervals derived from a multivariable linear regression are shown for binary outcomes, whereas regression coefficient and their 95%-confidence intervals computed with a multivariable linear regression are shown for the continuous outcome length of hospital stay. The associations are adjusted for age, gender and BMI. *P*-values are adjusted for multiple comparison for each outcome separately by means of the Bonferroni correction. .

Protein	Stroke		Myocardial infarction		1-year mortality		Length of stay	
	Incidence 12/192 (6.3%)		Incidence 6/192 (3.1%)		Incidence 5/192 (2.6%)		Median 7 [IQR: 6–9 ] days	
	OR (95%-CI)	*p* (adjusted *p*)	OR (95%-CI)	*p* (adjusted *p*)	OR (95%-CI)	*p* (adjusted *p*)	Beta (95%-CI)	*p* (adjusted *p*)
ACTA1	1.72 (0.82–3.70)	0.15 (>0.99)	0.98 (0.24–3.29)	0.97 (>0.99)	2.04 (0.81–5.11)	0.12 (>0.99)	0.25 (−1.14–1.64)	0.72 (>0.99)
ACTB;ACTG1	1.64 (0.82–3.39)	0.16 (>0.99)	0.96 (0.23–3.28)	0.95 (>0.99)	3.31 (1.32–8.93)	0.011 (0.32)	0.76 (−0.37–1.90)	0.19 (>0.99)
AGT	0.47 (0.14–1.48)	0.21 (>0.99)	0.79 (0.13–5.10)	0.80 (>0.99)	1.82 (0.29–12.16)	0.52 (>0.99)	−1.63 (−3.44–0.18)	0.08 (>0.99)
ALDOA	1.24 (0.48–2.55)	0.60 (>0.99)	1.07 (0.43–2.15)	0.86 (>0.99)	2.00 (0.84–5.76)	0.12 (>0.99)	0.98 (−0.31–2.27)	0.14 (>0.99)
C1S	0.89 (0.40–1.98)	0.78 (>0.99)	0.94 (0.34–2.59)	0.91 (>0.99)	0.85 (0.30–2.40)	0.77 (>0.99)	0.14 (−0.87–1.16)	0.78 (>0.99)
CAT	1.33 (0.79–2.32)	0.28 (>0.99)	2.01 (0.63–7.04)	0.24 (>0.99)	1.76 (0.71–4.70)	0.22 (>0.99)	−0.40 (−1.32–0.52)	0.39 (>0.99)
CD14	1.61 (0.53–4.56)	0.38 (>0.99)	0.90 (0.17–3.79)	0.89 (>0.99)	1.03 (0.19–4.28)	0.97 (>0.99)	1.02 (−0.50–2.53)	0.19 (>0.99)
CRP	0.99 (0.22–4.70)	0.99 (>0.99)	0.42 (0.02–4.91)	0.50 (>0.99)	1.00 (0.13–7.79)	>0.99 (>0.99)	0.21 (−1.95–2.37)	0.85 (>0.99)
CST3	0.82 (0.38–1.52)	0.58 (>0.99)	0.76 (0.24–2.09)	0.61 (>0.99)	1.48 (0.73–2.55)	0.17 (>0.99)	0.82 (−0.04–1.67)	0.06 (>0.99)
GAPDH	1.62 (0.84–3.14)	0.15 (>0.99)	0.79 (0.40–1.88)	0.54 (>0.99)	1.39 (0.65–3.40)	0.48 (>0.99)	0.52 (−0.19–1.23)	0.15 (>0.99)
GSTO1	2.24 (1.01–5.18)	0.05 (>0.99)	2.46 (0.53–12.31)	0.24 (>0.99)	3.85 (1.27–14.09)	0.021 (0.62)	0.50 (−0.80–1.81)	0.45 (>0.99)
IGFBP2	1.23 (0.66–1.94)	0.42 (>0.99)	0.90 (0.20–1.93)	0.84 (>0.99)	1.83 (1.16–3.06)	0.009 (0.26)	0.96 (0.18–1.74)	0.016 (0.48)
LBP	0.51 (0.06–3.44)	0.51 (>0.99)	0.38 (0.04–2.66)	0.34 (>0.99)	0.29 (0.01–3.79)	0.38 (>0.99)	0.09 (−2.27–2.44)	0.94 (>0.99)
LCP1	0.84 (0.38–1.85)	0.67 (>0.99)	0.82 (0.18–3.62)	0.80 (>0.99)	2.08 (0.68–5.95)	0.18 (>0.99)	−0.66 (−1.77–0.46)	0.25 (>0.99)
LDHB	1.33 (0.55–2.75)	0.48 (>0.99)	2.23 (0.93–6.13)	0.09 (>0.99)	2.51 (1.07–5.80)	0.024 (0.71)	0.63 (−0.53–1.78)	0.29 (>0.99)
LRG1	0.70 (0.12–3.41)	0.67 (>0.99)	0.21 (0.01–2.12)	0.22 (>0.99)	0.21 (0.02–2.02)	0.21 (>0.99)	1.29 (−0.68–3.27)	0.20 (>0.99)
MAN1A1	1.16 (0.36–3.42)	0.79 (>0.99)	0.74 (0.12–3.88)	0.73 (>0.99)	0.49 (0.09–2.45)	0.40 (>0.99)	0.72 (−0.83–2.27)	0.36 (>0.99)
PCSK9	0.75 (0.29–1.99)	0.56 (>0.99)	0.98 (0.41–2.62)	0.97 (>0.99)	0.77 (0.28–2.37)	0.62 (>0.99)	−0.51 (−1.52–0.50)	0.32 (>0.99)
PDE4DIP	1.53 (1.03–2.43)	0.048 (>0.99)	0.44 (0.16–1.03)	0.07 (>0.99)	0.88 (0.49–1.55)	0.67 (>0.99)	0.66 (−0.08–1.40)	0.08 (>0.99)
PFN1	1.25 (0.46–3.56)	0.67 (>0.99)	1.41 (0.31–7.47)	0.67 (>0.99)	1.84 (0.43–9.32)	0.43 (>0.99)	0.48 (−1.12–2.07)	0.56 (>0.99)
S100A8	1.24 (0.87–2.16)	0.30 (>0.99)	0.90 (0.16–1.75)	0.87 (>0.99)	1.88 (1.16–3.65)	0.022 (0.67)	−0.56 (−1.37–0.26)	0.18 (>0.99)
SAA1	0.63 (0.14–2.48)	0.53 (>0.99)	0.91 (0.12–6.34)	0.92 (>0.99)	0.19 (0.02–1.61)	0.16 (>0.99)	−0.57 (−2.45–1.31)	0.55 (>0.99)
SAA4	0.76 (0.35–1.74)	0.49 (>0.99)	0.75 (0.25–2.27)	0.60 (>0.99)	0.45 (0.16–1.34)	0.13 (>0.99)	−0.15 (−1.32–1.02)	0.8 (>0.99)
SERPINA1	0.23 (0.03–1.30)	0.11 (>0.99)	1.48 (0.17–13.56)	0.72 (>0.99)	0.36 (0.03–3.38)	0.39 (>0.99)	−0.74 (−3.04–1.56)	0.53 (>0.99)
SERPINA10	0.25 (0.04–1.35)	0.12 (>0.99)	0.16 (0.01–2.11)	0.19 (>0.99)	0.57 (0.06–5.44)	0.62 (>0.99)	−0.26 (−2.50–1.98)	0.82 (>0.99)
SERPINA3	0.36 (0.05–2.41)	0.30 (>0.99)	0.74 (0.16–3.62)	0.69 (>0.99)	0.47 (0.04–4.57)	0.54 (>0.99)	−0.04 (−2.24–2.15)	0.97 (>0.99)
SERPINA5	1.97 (0.44–9.04)	0.37 (>0.99)	0.81 (0.09–5.95)	0.84 (>0.99)	10.67 (1.31–115.38)	0.034 (>0.99)	−0.19 (−2.12–1.74)	0.85 (>0.99)
SPP2	1.02 (0.44–2.50)	0.97 (>0.99)	1.10 (0.28–5.56)	0.90 (>0.99)	0.89 (0.27–3.42)	0.86 (>0.99)	−0.05 (−1.42–1.31)	0.94 (>0.99)
VCAM1	0.73 (0.33–1.57)	0.43 (>0.99)	2.74 (0.66–13.11)	0.17 (>0.99)	0.52 (0.17–1.49)	0.24 (>0.99)	−0.33 (−1.41–0.75)	0.55 (>0.99)
VCL	2.54 (1.13–6.10)	0.025 (0.76)	0.70 (0.11–3.68)	0.70 (>0.99)	2.06 (0.86–4.70)	0.08 (>0.99)	0.65 (−0.63–1.94)	0.32 (>0.99)

### 3.4. Discussion

Through our innovative multi-step approach, we were able to identify a small subset of proteins within the acute phase proteome, which show relevant associations with clinical outcome measures, and are therefore promising candidates for pre- and perioperative acute phase response monitoring and potentially risk stratification.

As is the case in our patients with CPB, major changes to the serum proteome from the day of induction of anaesthesia to later postoperative stages have been described in cohorts involving individuals undergoing veno-arterial extracorporeal membrane oxygenation (22). In this study, three hit-proteins showed the strongest associations with clinical outcomes, VCAM1, IGFBP2 and LDHB, while the SERPINs yielded a large number of associations with outcome measures.

The SERPINs are known to play an important role in cardiovascular disease (23, 24). They are involved in the finely tuned balance between procoagulant and anticoagulant systems, due to their anticoagulant and antifibrinolytic properties and have even been proposed as a potential therapeutic target (25). Further, SERPINA1 is involved in the immune response by inhibiting ATP-induced interleukin-ß-release (26) and secreted into the bloodstream in response to myocardial infarction (27). Overall, SERPINA1 regulates the expression of chemokines, chemotaxis and cell adhesion and reduces the expression of pro-inflammatory cytokines and up-regulates anti-inflammatory mediators (28). Previous studies have even investigated SERPINA1 augmentation therapy during heart surgery to attenuate postoperative inflammation (26). Both SERPINA3 and SERPINA5 were detected in RNA sequencing of failing right ventricles and proposed as biomarkers for abnormalities in the involved inflammatory processes (29). In another study, SERPINA3 was identified as a potential predictive marker of clinical outcome after myocardial infarction, as the level of this protease inhibitor was found to be directly correlated with other measured inflammatory markers (30). The relevance of the observed association of uncorrected preoperative values with several clinical outcome measures in our study remains to be investigated—speculatively, the SERPINs might serve as a marker of subtle preoperative inflammation and cardiovascular stress.

VCAM1, just as SERPINA1, is also involved in inflammatory processes and is activated by tumor necrosis factor alpha and mediates vascular adhesion and trans-endothelial migration of leukocytes (31). This is also the case in myocardial injury, where VCAM1 has been shown to mediate rapid neutrophil mobilization (32). Therefore, the dynamics in VCAM1 levels observed in our study, might be attributed to perioperative myocardial injury and the consecutive inflammatory response. This is important as its serves as a potential target and has been proposed for the treatment of immune disease including autoimmune myocarditis or cancer (33). Furthermore, considering the association of preoperative levels with clinical outcome, VCAM1 has been suggested as a predictive biomarker for heart failure-related mortality and morbidity, endothelial injury in coronary artery disease and arrhythmias such as atrial fibrillation (33). This is also true for the perioperative setting, where an association of high preoperative VCAM1 levels with long-term (median follow-up of 6.7 years) all-cause mortality could be found in patients suffering from cardiovascular disease undergoing on-pump cardiac surgery. This association was independent of inflammatory markers and other cardiovascular risk factors (34). Contrarily, preoperative VCAM1 levels were associated with length of hospital stay but not mortality in our cohort.

IGFPB2 has also been suggested for cardiovascular risk monitoring (35). Its plasma concentration can be used to not only detect heart failure but also distinguish healthy individuals from patients with stable chronic disease. Thus, its diagnostic and prognostic value in heart failure especially when compared to natriuretic peptides is highly promising (36). Further, an early activation of IGFBP2 represents a marker of early smooth muscle cell phenotype modulation in patients suffering from thoracic aortic aneurysms (37). After acute MI, higher IGFPB2-levels were prognostic for higher risks of major adverse cardiac events after discharge (33). Of note, inflammation was shown to be a modulator of the insulin-like growth factor binding protein system in several pathological conditions (38, 39). In light of those findings, it is highly likely, that the association of preoperative IGFBP2 with all-cause mortality is a marker of a higher burden of cardiovascular disease at baseline and that the dynamics during cardiac surgery probably represent further myocardial injury.

The body of evidence confirms that our hit approach led to a conclusive selection of proteins, which are relevant to the dynamics of cardiovascular disease and are heavily altered through the exposition to surgical trauma and CPB. Overall, the identified proteins are likely to play an important role in the acute phase response to cardiac surgery due to their known biological functions in vascular function, coagulation, immune system homeostasis, tissue damage, and hypoxic stress. The inflammatory response to CPB is distinctively different from off-pump surgical trauma, resulting in an upregulation of apoptosis and remodeling markers (40). As shown by Ghorbel et al., cytokines and chemokines after CPB were elevated both at the mRNA level in the myocardium and at the protein level in the blood, suggesting the myocardium as likely source for these proteomic changes (40). The link between myocardial injury, ischemia-reperfusion and inflammation has been extensively investigated (41). While most of the identified proteins are not a direct component of the immune system, their exhibition during myocardial injury together with the interactions with the immune system described above, might reflect the interaction of perioperative myocardial injury with the acute phase response—however, this remains speculative. Most importantly, we cannot deduct whether the dynamics in the perioperative levels of these proteins should be attributed to myocardial injury, the exposition to CPB or a combination of the two. As prophylactically addressing inflammation in cardiac surgery has not shown any advantages (42, 43), a better understanding of the involved pathways during perioperative myocardial injury and the interaction with the acute phase response is crucial to potentially identify new therapeutic targets.

The most established and probably most investigated acute phase protein C-reactive protein (CRP) was also included in our selection of hit proteins. Preoperative CRP levels have been repeatedly shown to be an important determinant of short- and long-term postoperative outcome after on-pump cardiac surgery (44, 45). However, this was not the case in our cohort. Overall, baseline values of the hit-proteins identified in our study seemed to have stronger associations with outcome compared to perioperative change, which might suggest that the preoperative state of the investigated proteins is a stronger determinant of outcome than the perioperative response. Similarly, in contrast to the preoperative levels mentioned above, postoperative CRP levels did not seem be useful for predicting postoperative outcome in several studies (46, 47).

Accordingly, after adjustment for multiple comparison, the associations of preoperative levels of IGFBP2 with 1-year all-cause mortality, and the associations of preoperative levels of LDHB and VCAM1 with length of hospital stay remained significant, while no association of perioperative change of any protein with clinical outcome measures could be observed. As IGFBP2 and VCAM1 have been described above, measuring LDH has traditionally been used as an indicator of myocardial damage or necrosis and has been found to be elevated in patients suffering from valve heart disease, heart failure, and coronary heart disease (48). Levels of preoperative IGFB2 and LDHB were significantly associated with EuroSCORE II (19) at baseline in our cohort, indicating that those patients were probably in a more severe or progressed disease state.

Hence, the identified markers showed potential as prognostic markers before cardiac surgery. Creating combined scores consisting of multiple biomarkers might be a promising new approach for predicting outcome after cardiac surgery and our findings yielded potential candidates for further evaluation. As a most prominent example, the introduction of the cardiac-specific biomarkers natriuretic peptides and cardiac troponins have substantially refined the prognostication of cardiovascular risk in non-cardiac surgery, both independently and complementary to other important indicators of risk (49). Other recent studies have assessed the predictive power of other multimodal scores consisting of hit-proteins and previously identified risk factors such as age, haemoglobin values or serum lactate concentrations to predict neurologic outcomes in emergency patients undergoing cardiac surgery after out-of-hospital cardiac arrest (50). In paediatric patients undergoing on-pump cardiac surgery, a holistic approach to outcome prediction has previously been applied by creating a potential predictor model involving 24 key proteins with significant changes along the perioperative time course (51).

This study has several limitations. The observational design of the study prevents from inferring causal relationship. We did not exclude patients with autoimmune disease or infection. However, a clinically relevant infection is a contraindication for elective heart surgery at our center, which was also represented by the low CRP levels measured at baseline measured by routine laboratory methods, suggesting that the overall preoperative inflammatory status of our patients was low. Further, spectral counting is commonly used for identification and quantitative analysis in proteomics (52). This method has a tendency to mask low-abundance proteins, which are below the detection limit for mass spectrometers (53). Spectral counting allows to indirectly quantify protein levels, but does not represent their biological activity and exact functionality within a complex interrelated system. Further, while our multi-step approach allowed to identify hit-proteins, this methodology might also introduce a selection bias. Most importantly, due to the rigorous predefined selection process, proteins which only emerge during or after surgery or which are only present in a small subset of patients were not included in this analysis. We acknowledge, that those proteins might also play a crucial role in the perioperative acute phase response to cardiac surgery. Further, our analysis protocol led to a very small number of actual hit-proteins. While many of these showed significant associations with clinical outcome measures, an even smaller number of associations remained significant after correcting for multiple testing. However, this correction is essential, as the mass of data generated, might otherwise produce significance by chance. Once again, other important proteins might not have been included in our selection. Overall, despite being clinically motivated, the empirical choices inherent in our multi-step procedures—e.g., the definition of the ellipse when assessing the perioperative changes and the between-patient variation in perioperative protein changes jointly—might be chosen differently.

In conclusion, we were able to identify a subset of promising candidate proteins relevant to outcome after on-pump cardiac surgery, through applying an innovative multi-step approach. IGFBP2 yielded the most promising results with a strong association with clinical outcome measures and a significant association of preoperative levels with 1-year all-cause mortality. Further proteins with clinically relevant associations were LDHB and VCAM1. We recommend further investigation of these proteins as potential outcome markers after cardiac surgery.

## Data Availability

The datasets presented in this study can be found in online repositories. The names of the repository/repositories and accession number(s) can be found below: https://www.ebi.ac.uk/pride/archive/projects/PXD046496.

## References

[1] Rossaint J Zarbock A. Perioperative inflammation and its modulation by anesthetics. Anesth Analg. (2018) 126(3):1058–67. 10.1213/ANE.0000000000002484 28922235

[2] Pfortmueller CA Meisel C Fux M Schefold JC. Assessment of immune organ dysfunction in critical illness: utility of innate immune response markers. Intensive Care Med Exp. (2017) 5(1):49. 10.1186/s40635-017-0163-0 29063386 PMC5653680

[3] Butler J Rocker GM Westaby S. Inflammatory response to cardiopulmonary bypass. Ann Thorac Surg. (1993) 55(2):552–9. 10.1016/0003-4975(93)91048-R 8431082

[4] Hausenloy DJ Yellon DM. Ischaemic conditioning and reperfusion injury. Nat Rev Cardiol. (2016) 13(4):193–209. 10.1038/nrcardio.2016.5 26843289

[5] Arrell DK Neverova I Eyk JEV. Cardiovascular proteomics. Circ Res. (2001) 88(8):763–73. 10.1161/hh0801.090193 11325867

[6] Ravuri HG Sadowski P Noor Z Satake N Mills PC. Plasma proteomic changes in response to surgical trauma and a novel transdermal analgesic treatment in dogs. J Proteomics. (2022) 265:104648. 10.1016/j.jprot.2022.104648 35691609

[7] Cardinale F Chinellato I Caimmi S Peroni DG Franceschini F Miraglia Del Giudice M Perioperative period: immunological modifications. Int J Immunopathol Pharmacol. 2011;24(3 Suppl):S3–12. 10.1177/03946320110240S302 22014920

[8] Keel M Trentz O. Pathophysiology of polytrauma. Injury. (2005) 36(6):691–709. 10.1016/j.injury.2004.12.037 15910820

[9] Menger MD Vollmar B. Surgical trauma: hyperinflammation versus immunosuppression? Langenbecks Arch Surg. (2004) 389(6):475–84. 10.1007/s00423-004-0472-0 15173946

[10] David P Hansen FJ Bhat A Weber GF. An overview of proteomic methods for the study of “cytokine storms”. Expert Rev Proteomics. (2021) 18(2):83–91. 10.1080/14789450.2021.1911652 33849358

[11] Hogan BV Peter MB Shenoy HG Horgan K Hughes TA. Surgery induced immunosuppression. Surgeon. (2011) 9(1):38–43. 10.1016/j.surge.2010.07.011 21195330

[12] Dobson GP. Trauma of major surgery: a global problem that is not going away. Int J Surg. (2020) 81:47–54. 10.1016/j.ijsu.2020.07.017 32738546 PMC7388795

[13] Noble WS MacCoss MJ. Computational and statistical analysis of protein mass spectrometry data. PLoS Comput Biol. (2012) 8(1):e1002296. 10.1371/journal.pcbi.1002296 22291580 PMC3266873

[14] Heinisch PP Mihalj M Huber M Schefold JC Hartmann A Walter M Impact of lipoprotein(a) levels on perioperative outcomes in cardiac surgery. Cells. (2021) 10(11):2829. 10.3390/cells10112829 34831051 PMC8616553

[15] Mihalj M Heinisch PP Huber M Schefold JC Hartmann A Walter M Effect of perioperative lipid status on clinical outcomes after cardiac surgery. Cells. (2021) 10(10):2717. 10.3390/cells10102717 34685697 PMC8534806

[16] Heinisch PP Mihalj M Haliguer E Gahl B Winkler B Venetz P Initial experience with minimally invasive extracorporeal circulation in coronary artery bypass graft reoperations. Swiss Med Wkly. (2022) 152:w30101. 10.4414/SMW.2022.w30101 35195525

[17] Perez-Riverol Y Bai J Bandla C Garcia-Seisdedos D Hewapathirana S Kamatchinathan S The pride database resources in 2022: a hub for mass spectrometry-based proteomics evidences. Nucleic Acids Res. (2022) 50(D1):D543–52. 10.1093/nar/gkab1038 34723319 PMC8728295

[18] Thygesen K Alpert JS Jaffe AS Chaitman BR Bax JJ Morrow DA Fourth universal definition of myocardial infarction. J Am Coll Cardiol. (2018) 72(18):2231–64. 10.1016/j.jacc.2018.08.1038 30153967

[19] Nashef SAM Roques F Sharples LD Nilsson J Smith C Goldstone AR Euroscore II. Eur J Cardiothorac Surg. (2012) 41(4):734–45. 10.1093/ejcts/ezs043 22378855

[20] Kyoto Encyclopedia of Genes and Genomes. Available at: https://www.genome.jp/kegg/pathway.html

[21] Team RDC. R: A Language and Environment for Statistical Computing. (No Title). (2010).

[22] Siegel PM Barta BA Orlean L Steenbuck ID Cosenza-Contreras M Wengenmayer T The serum proteome of VA-ECMO patients changes over time and allows differentiation of survivors and non-survivors: an observational study. J Transl Med. (2023) 21(1):319. 10.1186/s12967-023-04174-8 37173738 PMC10176307

[23] Sánchez-Navarro A González-Soria I Caldiño-Bohn R Bobadilla NA. An integrative view of serpins in health and disease: the contribution of serpinA3. Am J Physiol Cell Physiol. (2021) 320(1):C106–18. 10.1152/ajpcell.00366.2020 33112643

[24] Crawford AA Bankier S Altmaier E Barnes CLK Clark DW Ermel R Variation in the SERPINA6/SERPINA1 locus alters morning plasma cortisol, hepatic corticosteroid binding globulin expression, gene expression in peripheral tissues, and risk of cardiovascular disease. J Hum Genet. (2021) 66(6):625–36. 10.1038/s10038-020-00895-6 33469137 PMC8144017

[25] Bouton MC Corral J Lucas AR. Editorial: the serpin family in the cardiovascular system. Front Cardiovasc Med. (2021) 8:821490. 10.3389/fcvm.2021.821490 35187116 PMC8847448

[26] Agné A Richter K Tumpara S Sauer AL Beckert F Wrenger S Does heart surgery change the capacity of *α*1-antitrypsin to inhibit the ATP-induced release of monocytic interleukin-1β? A preliminary study. Int Immunopharmacol. (2020) 81:106297. 10.1016/j.intimp.2020.106297 32062078

[27] Meijers WC Maglione M Bakker SJL Oberhuber R Kieneker LM de Jong S Heart failure stimulates tumor growth by circulating factors. Circulation. (2018) 138(7):678–91. 10.1161/CIRCULATIONAHA.117.030816 29459363

[28] Bergin DA Reeves EP Meleady P Henry M McElvaney OJ Carroll TP *α*-1 antitrypsin regulates human neutrophil chemotaxis induced by soluble immune complexes and IL-8. J Clin Invest. (2010) 120(12):4236–50. 10.1172/JCI41196 21060150 PMC2993580

[29] di Salvo TG Yang KC Brittain E Absi T Maltais S Hemnes A. Right ventricular myocardial biomarkers in human heart failure. J Card Fail. (2015) 21(5):398–411. 10.1016/j.cardfail.2015.02.005 25725476 PMC6482959

[30] Zhao L Zheng M Guo Z Li K Liu Y Chen M Circulating Serpina3 levels predict the major adverse cardiac events in patients with myocardial infarction. Int J Cardiol. (2020) 300:34–8. 10.1016/j.ijcard.2019.08.034 31439424

[31] Kong D-H Kim YK Kim MR Jang JH Lee S. Emerging roles of vascular cell adhesion molecule-1 (VCAM-1) in immunological disorders and cancer. Int J Mol Sci. (2018) 19(4):1057. 10.3390/ijms19041057 29614819 PMC5979609

[32] Akbar N Braithwaite AT Corr EM Koelwyn GJ van Solingen C Cochain C Rapid neutrophil mobilization by VCAM-1+endothelial cell-derived extracellular vesicles. Cardiovasc Res. (2023) 119(1):236–51. 10.1093/cvr/cvac012 35134856 PMC10022859

[33] Troncoso MF Ortiz-Quintero J Garrido-Moreno V Sanhueza-Olivares F Guerrero-Moncayo A Chiong M VCAM-1 as a predictor biomarker in cardiovascular disease. Biochim Biophys Acta Mol Basis Dis. (2021) 1867(9):166170. 10.1016/j.bbadis.2021.166170 34000374

[34] Corbalan R Garcia M Garrido-Olivares L Garcia L Perez G Mellado R Preoperative soluble VCAM-1 contributes to predict late mortality after coronary artery surgery. Clin Cardiol. (2020) 43(11):1301–7. 10.1002/clc.23443 32770579 PMC7661653

[35] Heald AH Kaushal K Siddals KW Rudenski AS Anderson SG Gibson JM. Insulin-like growth factor binding protein-2 (IGFBP-2) is a marker for the metabolic syndrome. Exp Clin Endocrinol Diabetes. (2006) 114(7):371–6. 10.1055/s-2006-924320 16915540

[36] Berry M Galinier M Delmas C Fournier P Desmoulin F Turkieh A Proteomics analysis reveals IGFBP2 as a candidate diagnostic biomarker for heart failure. IJC Metab Endocr. (2015) 6:5–12. 10.1016/j.ijcme.2014.11.003

[37] Pedroza AJ Dalal AR Shad R Cheng P Yokoyama N Nakamura K Abstract 11990: insulin-like growth factor binding protein 2 is a novel marker of early smooth muscle cell phenotype modulation in thoracic aortic aneurysm. Circulation. (2021) 144(Suppl_1):A11990-A. 10.1161/circ.144.suppl_1.12006

[38] Street ME Miraki-Moud F Sanderson IR Savage MO Giovannelli G Bernasconi S Interleukin-1beta (IL-1beta) and IL-6 modulate insulin-like growth factor-binding protein (IGFBP) secretion in colon cancer epithelial (caco-2) cells. J Endocrinol. (2003) 179(3):405–15. 10.1677/joe.0.1790405 14656210

[39] Street ME Ziveri MA Spaggiari C Viani I Volta C Grzincich GL Inflammation is a modulator of the insulin-like growth factor (IGF)/IGF-binding protein system inducing reduced bioactivity of IGFs in cystic fibrosis. Eur J Endocrinol. (2006) 154(1):47–52. 10.1530/eje.1.02064 16381990

[40] Ghorbel MT Cherif M Mokhtari A Bruno VD Caputo M Angelini GD. Off-pump coronary artery bypass surgery is associated with fewer gene expression changes in the human myocardium in comparison with on-pump surgery. Physiol Genomics. (2010) 42(1):67–75. 10.1152/physiolgenomics.00174.2009 20332183 PMC2888559

[41] Algoet M Janssens S Himmelreich U Gsell W Pusovnik M Van den Eynde J Myocardial ischemia-reperfusion injury and the influence of inflammation. Trends Cardiovasc Med. (2023) 33(6):357–66. 10.1016/j.tcm.2022.02.005 35181472

[42] Squiccimarro E Stasi A Lorusso R Paparella D. Narrative review of the systemic inflammatory reaction to cardiac surgery and cardiopulmonary bypass. Artif Organs. (2022) 46(4):568–77. 10.1111/aor.14171 35061922 PMC9303696

[43] Whitlock RP Devereaux PJ Teoh KH Lamy A Vincent J Pogue J Methylprednisolone in patients undergoing cardiopulmonary bypass (SIRS): a randomised, double-blind, placebo-controlled trial. Lancet. (2015) 386(10000):1243–53. 10.1016/S0140-6736(15)00273-1 26460660

[44] Kangasniemi OP Biancari F Luukkonen J Vuorisalo S Satta J Pokela R Preoperative C-reactive protein is predictive of long-term outcome after coronary artery bypass surgery. Eur J Cardiothorac Surg. (2006) 29(6):983–5. 10.1016/j.ejcts.2006.02.022 16682213

[45] Balciunas M Bagdonaite L Samalavicius R Griskevicius L Vuylsteke A. Pre-operative high sensitive C-reactive protein predicts cardiovascular events after coronary artery bypass grafting surgery: a prospective observational study. Ann Card Anaesth. (2009) 12(2):127–32. 10.4103/0971-9784.53442 19602737

[46] Corral L Carrió ML Ventura JL Torrado H Javierre C Rodriguez-Castro D Is C-reactive protein a biomarker for immediate clinical outcome after cardiac surgery? J Cardiothorac Vasc Anesth. (2009) 23(2):166–9. 10.1053/j.jvca.2008.11.014 19201207

[47] Boralessa H de Beer FC Manchie A Whitwam JG Pepys MB. C-reactive protein in patients undergoing cardiac surgery. Anaesthesia. (1986) 41(1):11–5. 10.1111/j.1365-2044.1986.tb12696.x 3946770

[48] Zeng Y Zhao Y Dai S Liu Y Zhang R Yan H Impact of lactate dehydrogenase on prognosis of patients undergoing cardiac surgery. BMC Cardiovasc Disord. (2022) 22(1):404. 10.1186/s12872-022-02848-7 36088306 PMC9463775

[49] Clerico A Zaninotto M Aimo A Musetti V Perrone M Padoan A Evaluation of the cardiovascular risk in patients undergoing major non-cardiac surgery: role of cardiac-specific biomarkers. Clin Chem Lab Med. (2022) 60(10):1525–42. 10.1515/cclm-2022-0481 35858238

[50] Distelmaier K Muqaku B Wurm R Arfsten H Seidel S Kovacs GG Proteomics-enriched prediction model for poor neurologic outcome in cardiac arrest survivors. Crit Care Med. (2020) 48(2):167–75. 10.1097/CCM.0000000000004105 31939784

[51] Umstead TM Lu CJ Freeman WM Myers JL Clark JB Thomas NJ The kinetics of cardiopulmonary bypass: a dual-platform proteomics study of plasma biomarkers in pediatric patients undergoing cardiopulmonary bypass. Artif Organs. (2012) 36(1):E1–20. 10.1111/j.1525-1594.2011.01412.x 22250822

[52] Choi H Fermin D Nesvizhskii AI. Significance analysis of spectral count data in label-free shotgun proteomics. Mol Cell Proteomics. (2008) 7(12):2373–85. 10.1074/mcp.M800203-MCP200 18644780 PMC2596341

[53] Lundgren DH Hwang S-I Wu L Han DK. Role of spectral counting in quantitative proteomics. Expert Rev Proteomics. (2010) 7(1):39–53. 10.1586/epr.09.69 20121475

