# Supplementary material for: Discovery of plasma proteome markers associated with clinical outcome and immunological stress after cardiac surgery
Source: Front Cardiovasc Med. 2023 Dec 22;10:1287724. doi: 10.3389/fcvm.2023.1287724 (PMC10876477; doi:10.3389/fcvm.2023.1287724)

Supplementary Material

# Supplementary Data

**Supplementary Data Table 1.** List of all acute phase proteins detected.

| **Genes** | **Median [IQR]** | **Genes** | **Median [IQR]** | **Genes** | **Median [IQR]** | **Genes** | **Median [IQR]** | **Genes** | **Median [IQR]** | **Genes** | **Median [IQR]** |
| --- | --- | --- | --- | --- | --- | --- | --- | --- | --- | --- | --- |
| A1BG | 437903 [394367 - 504892] | CD163 | 2105 [1664 - 2543] | FGG | 932123 [817991 - 1110000] | IGHV3-43 | 4051 [3241 - 5379] | IGLV9-49 | 8634 [5987 - 11552] | POSTN | 1751 [1482 - 2215] |
| A2M | 2060000 [1687500 - 2472500] | CD5L | 70307 [51530 - 92577] | FGL1 | 1774 [1296 - 2548] | IGHV3-43D | 32323 [22609 - 43373] | IGSF1 | 574 [409 - 787] | PPBP | 5092 [3909 - 6365] |
| ABCC2 | 10959 [6092 - 19603] | CDH1 | 1232 [878 - 1565] | FLNA | 2898 [2244 - 4138] | IGHV3-49 | 261478 [200593 - 327229] | IL1RAP | 6066 [4146 - 8393] | PPIA | 2261 [1938 - 2698] |
| ACTA1 | 3041 [2187 - 4158] | CDH5 | 3618 [2766 - 4365] | FN1 | 30505 [21332 - 51432] | IGHV3-64 | 12865 [9468 - 18133] | ITIH1 | 238925 [213317 - 270346] | PRDX2 | 3222 [2094 - 4318] |
| ACTB;ACTG1 | 15966 [12565 - 19866] | CETP | 6258 [5274 - 8005] | FTL | 2776 [1862 - 3889] | IGHV3-64D | 12844 [8230 - 17226] | ITIH2 | 898892 [795625 - 996028] | PRDX6 | 3118 [2565 - 3858] |
| ACTBL2 | 7854 [4245 - 10436] | CFB | 377640 [340834 - 430184] | GAPDH | 10341 [8187 - 13004] | IGHV3-7 | 637202 [495318 - 775211] | ITIH3 | 59473 [47449 - 74440] | PRG2 | 2500 [1926 - 3171] |
| ACTN4 | 2559 [2129 - 3300] | CFD | 22843 [19545 - 28176] | GC | 435537 [380461 - 513253] | IGHV3-72 | 15023 [10847 - 19844] | ITIH4 | 458413 [417376 - 519904] | PRG4 | 6993 [5879 - 8955] |
| ADH1B | 2574 [2227 - 3099] | CFH | 525442 [476856 - 573773] | GGH | 2948 [2175 - 3586] | IGHV3-73 | 5818 [4423 - 8064] | JCHAIN | 88989 [69160 - 123619] | PROC | 11650 [9863 - 13243] |
| ADIPOQ | 9158 [6294 - 13287] | CFHR1 | 26144 [18947 - 33515] | GKN1 | 884 [691 - 1120] | IGHV3-74 | 7871 [5917 - 10972] | KLKB1 | 116900 [101292 - 132985] | PROCR | 2082 [1581 - 2752] |
| AFM | 152078 [111921 - 181748] | CFHR2 | 10908 [7055 - 15396] | GMDS | 804 [683 - 1466] | IGHV4-28 | 5491 [4159 - 7818] | KNG1 | 583084 [534300 - 643822] | PROS1 | 56564 [50877 - 62200] |
| AGT | 169582 [150535 - 192571] | CFHR4 | 15643 [10215 - 23758] | GP1BA | 2649 [1995 - 3325] | IGHV4-38-2 | 37862 [28418 - 51697] | KRT1 | 5374 [3723 - 9044] | PROZ | 7888 [5726 - 9738] |
| AHSG | 828883 [735350 - 927816] | CFHR5 | 4156 [3660 - 4765] | GPI | 628 [579 - 848] | IGHV4-4 | 9136 [6329 - 13286] | KRT10 | 2681 [1909 - 4037] | PRSS1 | 70914 [37223 - 155020] |
| ALAD | 3777 [2771 - 4330] | CFI | 124491 [113613 - 135507] | GPLD1 | 38115 [30636 - 45741] | IGHV5-51 | 65725 [47929 - 83965] | KRT14 | 1603 [948 - 2350] | PRTN3 | 1693 [1345 - 2315] |
| ALB | 5945000 [5420000 - 6750000] | CFL2 | 3124 [2530 - 3694] | GPX3 | 59984 [48223 - 68715] | IGHV6-1 | 3193 [2194 - 4580] | KRT16 | 889 [801 - 1499] | PSMG3-AS1 | 3332 [3135 - 4113] |
| Albumin | 621272 [517863 - 733548] | CFP | 21005 [17120 - 24889] | GSN | 227132 [194673 - 254824] | IGHV7-4-1 | 3054 [2501 - 4201] | KRT2 | 3255 [2579 - 4883] | PTGDS | 9988 [8100 - 12711] |
| ALDH1A1 | 2649 [1487 - 3080] | CHGA | 1960 [1477 - 2304] | GSTO1 | 2898 [2276 - 3548] | IGKC | 1215000 [961108 - 1497500] | KRT5 | 1808 [1555 - 2609] | PTX3 | NA [NA - NA] |
| ALDOA | 2626 [2174 - 3323] | CHI3L1 | 7425 [5786 - 10632] | GSTP1 | 428 [326 - 530] | IGKV1-12;IGKV1D-12 | 58561 [41555 - 82488] | KRT6A;KRT6C | 1496 [1071 - 2141] | PZP | 6453 [2834 - 14464] |
| ALDOB | 3784 [2769 - 5314] | CHL1 | 3786 [2975 - 4803] | H4C16 | 2276 [1475 - 2688] | IGKV1-13;IGKV1D-13 | 16834 [10860 - 30999] | KRT8 | 3730 [2505 - 6207] | QSOX1 | 11239 [9573 - 12935] |
| AMBP | 304554 [269926 - 350720] | CKM | 2059 [1569 - 3242] | HABP2 | 24955 [19728 - 29235] | IGKV1-16 | 8791 [6982 - 12364] | KRT9 | 4885 [3126 - 8981] | RARRES2 | 4893 [3749 - 6071] |
| AMY2A | 1213 [946 - 1433] | CLEC3B | 35245 [28740 - 42454] | HBA2 | 83996 [67919 - 115513] | IGKV1-17 | 20002 [15544 - 25663] | LBP | 25966 [20740 - 31231] | RBP4 | 138644 [101177 - 173392] |
| APCS | 238106 [190846 - 287843] | CLU | 301690 [276153 - 349856] | HBB | 186262 [140385 - 254413] | IGKV1-27 | 71056 [56412 - 87556] | LCAT | 21168 [18064 - 24936] | RNASE4 | 5407 [3963 - 7339] |
| APMAP | 7936 [6150 - 9786] | CNDP1 | 19079 [15268 - 22910] | HBD | 5912 [4351 - 9078] | IGKV1-33;IGKV1D-33 | 21647 [13305 - 39661] | LCN2 | 3635 [3022 - 4455] | RNH1 | 838 [550 - 1230] |
| APOA1 | 2545000 [2207500 - 2882500] | COL18A1 | 3473 [2948 - 4211] | HBG2 | 3922 [2767 - 5619] | IGKV1-37;IGKV1D-37 | 10260 [6374 - 17442] | LCP1 | 8087 [6883 - 9442] | RPS27A;UBA52;UBB;UBC | 1871 [1368 - 2828] |
| APOA2 | 178676 [140361 - 216513] | COL6A3 | 2223 [1583 - 3261] | HGFAC | 21719 [18372 - 25267] | IGKV1-39;IGKV1D-39 | 114256 [87716 - 136341] | LDHA | 765 [623 - 909] | S100A8 | 2980 [2125 - 4115] |
| APOA4 | 285018 [238540 - 349403] | COLEC11 | 1546 [1219 - 1945] | HLA-H | 1598 [1357 - 2590] | IGKV1-5 | 114540 [90163 - 142808] | LDHB | 3765 [3159 - 4422] | S100A9 | 1212 [957 - 1682] |
| APOB | 780248 [648805 - 890395] | COMP | 910 [721 - 1154] | HP | 3090000 [2390000 - 3772500] | IGKV1-8 | 9245 [6928 - 11945] | LGALS3BP | 34152 [28070 - 44539] | SAA1 | 8191 [5510 - 14149] |
| APOC1 | 103566 [84378 - 122026] | CP | 659102 [602283 - 730273] | HPR | 115784 [84635 - 149938] | IGKV1D-16 | 13100 [9852 - 17116] | LILRA3 | 662 [496 - 964] | SAA2 | 4242 [2577 - 7137] |
| APOC2 | 19002 [13252 - 24829] | CPA1 | 984 [674 - 5651] | HPX | 2115000 [1905000 - 2290000] | IGKV1D-8 | 11506 [8634 - 13860] | LPA | 25024 [11432 - 86410] | SAA4 | 237229 [186392 - 303588] |
| APOC3 | 157869 [121733 - 208037] | CPB2 | 26600 [22918 - 30377] | HRG | 378842 [319546 - 445856] | IGKV2-24;IGKV2D-24 | 87117 [64461 - 115359] | LRG1 | 53868 [41466 - 68840] | SELENBP1 | 3067 [2396 - 4164] |
| APOC4 | 13762 [9253 - 18718] | CPN1 | 42229 [36857 - 48294] | HSP90AA1 | 1337 [910 - 2247] | IGKV2-28;IGKV2D-28 | 530170 [405283 - 702989] | LTF | 19679 [11976 - 27875] | SELENOP | 20234 [16534 - 24846] |
| APOD | 178588 [144720 - 211380] | CPN2 | 77668 [67594 - 86752] | HSPA2 | 899 [635 - 1051] | IGKV2-30 | 4711 [3277 - 5793] | LUM | 49056 [38556 - 59208] | Serine | 1750000 [1087500 - 2262500] |
| APOE | 180323 [149850 - 221952] | CPS1 | 711 [647 - 917] | HSPA5 | 3230 [2742 - 3894] | IGKV2-40;IGKV2D-40 | 6041 [4842 - 8318] | LYZ | 6583 [4669 - 9498] | SERPINA1 | 1730000 [1567500 - 1950000] |
| APOH | 631326 [534661 - 730104] | CRISP3 | 3358 [2732 - 4085] | HSPA8 | 745 [531 - 909] | IGKV2D-29 | 6366 [4477 - 9705] | MAN1A1 | 5641 [4643 - 6435] | SERPINA10 | 14285 [12490 - 16501] |
| APOL1 | 131939 [116172 - 151936] | CRP | 9994 [4773 - 18861] | ICAM1 | 1058 [816 - 1357] | IGKV2D-30 | 2121 [1695 - 3042] | MASP1 | 10596 [8810 - 12550] | SERPINA3 | 1380000 [1230000 - 1592500] |
| APOM | 44180 [37502 - 51735] | CRTAC1 | 7696 [6325 - 9041] | IGF2 | 4933 [2978 - 7068] | IGKV3-11;IGKV3D-11 | 356952 [193370 - 602081] | MASP2 | 11373 [9991 - 13202] | SERPINA4 | 69428 [58737 - 77732] |
| ARSB | 70446 [46173 - 105241] | CST3 | 14249 [10603 - 17474] | IGFALS | 53665 [44086 - 64536] | IGKV3-20 | 252458 [193928 - 325891] | MB | 832 [619 - 933] | SERPINA5 | 21712 [18521 - 24730] |
| ATRN | 88115 [78604 - 97913] | CTRB1;CTRB2 | 6148 [3997 - 8323] | IGFBP2 | 2896 [2294 - 3734] | IGKV3-7 | 97692 [65792 - 126501] | MBL2 | 3929 [2957 - 5781] | SERPINA6 | 143739 [130549 - 163110] |
| AZGP1 | 230522 [200327 - 264401] | CTSB | 1756 [1397 - 2383] | IGFBP3 | 13588 [11018 - 17169] | IGKV3D-15 | 78108 [55614 - 108725] | MDH1 | 2090 [1266 - 3508] | SERPINA7 | 51587 [46137 - 58983] |
| B2M | 3700 [2835 - 4849] | CTSD | 1754 [1240 - 2170] | IGHA1 | 2665000 [1872500 - 3360000] | IGKV3D-20 | 48953 [26918 - 96261] | MINPP1 | 5627 [4419 - 6872] | SERPINB1 | 2074 [1531 - 2457] |
| BCHE | 53064 [44585 - 61335] | CTSG | 1260 [940 - 1876] | IGHA2 | 166998 [87396 - 310423] | IGKV4-1 | 1500000 [1107500 - 1960000] | MMP9 | 940 [497 - 1357] | SERPINC1 | 762716 [690231 - 827293] |
| BLVRB | 1615 [1279 - 1935] | DBH | 10018 [6620 - 14086] | IGHD | 39640 [20993 - 74480] | IGKV6-21 | 16112 [11902 - 21201] | MPO | 814 [696 - 1174] | SERPIND1 | 204772 [172199 - 225750] |
| BPGM | 1055 [871 - 1130] | DEFA1B;DEFA3 | 1862 [1339 - 2532] | IGHE | 3156 [2361 - 6301] | IGKV6D-41 | 4537 [3102 - 6925] | MST1 | 17684 [14848 - 19618] | SERPINE1 | 1782 [1600 - 2632] |
| BTD | 16591 [13028 - 19219] | ECM1 | 21418 [17750 - 26544] | IGHG1 | 3820000 [3222500 - 4480000] | IGLC1 | 15165 [9743 - 23392] | NME2 | 1969 [1338 - 3031] | SERPINF1 | 90752 [79713 - 99873] |
| C1QA | 39987 [34635 - 46511] | EFEMP1 | 7865 [6423 - 9648] | IGHG2 | 2560000 [1950000 - 3127500] | IGLC2 | 1940000 [1677500 - 2390000] | NRP1 | 2600 [2198 - 3141] | SERPINF2 | 178578 [165740 - 194230] |
| C1QB | 88385 [77487 - 98475] | ELANE | 737 [442 - 1074] | IGHG3 | 1070000 [830834 - 1372500] | IGLC7 | 1390 [704 - 3226] | ORM1 | 849076 [651969 - 1070000] | SERPING1 | 952590 [801345 - 1090000] |
| C1QC | 193098 [166556 - 216492] | ENO1 | 1900 [1559 - 2318] | IGHG4 | 717561 [375142 - 1090000] | IGLL1 | 36189 [24762 - 46654] | ORM2 | 13860 [10972 - 19307] | SHBG | 24628 [20137 - 33947] |
| C1R | 90562 [83542 - 98456] | F10 | 44885 [37276 - 52051] | IGHM | 916250 [654954 - 1135000] | IGLV1-36 | 8299 [3806 - 9163] | PAM | 1617 [1214 - 2220] | SOD3 | 2110 [1583 - 2968] |
| C1RL | 29361 [25209 - 33496] | F11 | 18300 [16034 - 20904] | IGHV1-18 | 18831 [14814 - 24556] | IGLV1-40 | 21345 [10737 - 34744] | PCOLCE | 4322 [3392 - 5361] | SPP2 | 7633 [5401 - 9648] |
| C1S | 75893 [69483 - 82145] | F12 | 50126 [42105 - 58907] | IGHV1-2 | 33590 [26158 - 46156] | IGLV1-47 | 87120 [50668 - 112200] | PCSK9 | 2258 [1721 - 2906] | TF | 3745000 [3390000 - 4172500] |
| C2 | 83144 [73909 - 89893] | F13A1 | 22393 [19882 - 25571] | IGHV1-24 | 7271 [4874 - 10818] | IGLV1-51 | 34394 [20309 - 55004] | PCYOX1 | 10840 [8679 - 13129] | TFRC | 50550 [42848 - 60609] |
| C3 | 2495000 [2247500 - 2692500] | F13B | 18741 [16054 - 22210] | IGHV1-3 | 13726 [10282 - 18651] | IGLV2-11 | 4472 [2641 - 8190] | PDE4DIP | 7036 [4489 - 11445] | TGFBI | 13029 [11364 - 15024] |
| C4A | 659152 [564100 - 781173] | F2 | 283068 [249577 - 310190] | IGHV1-45 | 24942 [18332 - 32512] | IGLV2-14 | 46941 [9058 - 70467] | PEBP1 | 1216 [952 - 1543] | THBS1 | 1835 [1470 - 2427] |
| C4B_2 | 134752 [98353 - 178373] | F5 | 22732 [20189 - 25504] | IGHV1-46 | 26287 [19531 - 37326] | IGLV2-18 | 29614 [18692 - 42842] | PF4 | 5492 [4364 - 6725] | THBS4 | 8033 [5963 - 10186] |
| C4BPA | 455586 [387057 - 544878] | F7 | 2954 [2516 - 3479] | IGHV1-58 | 2451 [1705 - 3348] | IGLV2-8 | 11152 [6791 - 23826] | PFN1 | 1822 [1315 - 2649] | TIMP1 | 3940 [3174 - 4584] |
| C4BPB | 12468 [10135 - 15217] | F9 | 30047 [27250 - 33262] | IGHV1-69 | 15143 [10056 - 22074] | IGLV3-10 | 12980 [8424 - 18963] | PGAM2 | NA [NA - NA] | TLN1 | 1428 [1023 - 1737] |
| C5 | 94541 [86384 - 103853] | FABP4;PMP2 | 446 [356 - 572] | IGHV1-8 | 9176 [6655 - 13519] | IGLV3-12 | 2429 [1781 - 3814] | PGD | 2562 [2367 - 3555] | TNXB | 2706 [2089 - 3204] |
| C6 | 76221 [67862 - 84256] | FAH | 1481 [1077 - 2012] | IGHV2-26 | 2383 [1735 - 3708] | IGLV3-19 | 7137 [4609 - 11904] | PGK1 | 891 [651 - 1661] | TPI1 | 1354 [1104 - 1630] |
| C7 | 82927 [70084 - 95496] | FAM91A1 | 92327 [80226 - 121083] | IGHV2-5 | 15033 [9661 - 20605] | IGLV3-21 | 10144 [7018 - 13633] | PGLYRP2 | 139196 [121792 - 157974] | TRAP1 | 934 [797 - 1097] |
| C8A | 81688 [73832 - 93392] | FBLN1 | 41615 [35371 - 49844] | IGHV2-70D | 4801 [3318 - 6581] | IGLV3-25 | 19570 [14053 - 25249] | PI16 | 4257 [3145 - 5389] | TRIM42 | 40659 [27746 - 58162] |
| C8B | 48145 [43744 - 53661] | FCGBP | 10440 [8484 - 13307] | IGHV3-13 | 9683 [7902 - 12580] | IGLV3-27 | 8534 [6272 - 10999] | PIGR | 4167 [3239 - 5562] | TTR | 825174 [681040 - 958876] |
| C8G | 53350 [48659 - 59053] | FCGR3A | 3915 [2936 - 4808] | IGHV3-15 | 19793 [15461 - 25781] | IGLV3-9 | 96842 [69437 - 131296] | PKM | 2691 [2077 - 3596] | VASN | 5475 [4643 - 6348] |
| C9 | 222882 [185430 - 263720] | FCN2 | 6378 [4618 - 8644] | IGHV3-20 | 879 [747 - 1073] | IGLV4-69 | 2543 [2047 - 3408] | PLG | 445215 [396595 - 477431] | VCAM1 | 1871 [1493 - 2150] |
| CA1 | 3560 [2741 - 4563] | FCN3 | 22506 [17654 - 27932] | IGHV3-30;IGHV3-30-5 | 432228 [361062 - 503664] | IGLV5-39 | 5528 [3435 - 8350] | PLIN1 | 28403 [16852 - 42775] | VCL | 1544 [1234 - 1951] |
| CA2 | 1113 [671 - 1775] | FERMT3 | 998 [721 - 1327] | IGHV3-33 | 2761 [2099 - 3212] | IGLV6-57 | 5075 [3037 - 7795] | PLTP | 12073 [10287 - 13604] | VTN | 447258 [366924 - 542762] |
| CARD9 | 10003 [6084 - 15721] | FETUB | 7080 [4767 - 11105] | IGHV3-35 | 62756 [42676 - 82788] | IGLV7-43 | 1816 [1402 - 3007] | PNLIP | 332 [332 - 332] | VWF | 10406 [8343 - 13547] |
| CAT | 2141 [1601 - 3042] | FGA | 880566 [768588 - 1035000] | IGHV3-38 | 54614 [40394 - 76094] | IGLV7-46 | 10648 [7802 - 14829] | PON1 | 256504 [214964 - 305507] | YWHAE | 1359 [1213 - 1376] |
| CD14 | 19358 [16545 - 22350] | FGB | 998768 [888144 - 1172500] | IGHV3-38-3 | 15209 [8830 - 22538] | IGLV8-61 | 31351 [19560 - 44875] | PON3 | 8622 [4905 - 21024] | YWHAZ | 1290 [1077 - 1652] |

**Supplementary Data Table 2.** Descriptive summary of the perioperative change in CRP values measured by conventional laboratory methods.

| **Biomarker** | **Preoperative***^a^* | **Post-operative***^a^* | **Change***^b^* |
| --- | --- | --- | --- |
| **CRP** (mg/L) | 3.0^c^ [3.0^c^; 3.2] | 77.0 [60.8; 99.0] | 73.5 (70.0 - 77.5) |

CRP = C-reactive protein

*^a^* Data are expressed as mean (IQR)

*^b^* The median and associated 95%-confidence interval of the paired difference (post-operative minus preoperative).

*^c^* This value refers to the rounded default value of 2.99 that was chosen for CRP values <3 mg/L.

## Supplementary Figures

**Supplementary Figure 1.** Unadjusted associations of centered and scaled preoperative protein levels with binary patient characteristics. Mean and 95%-confidence intervals of the odds ratios derived from univariable logistic regression are shown.

**
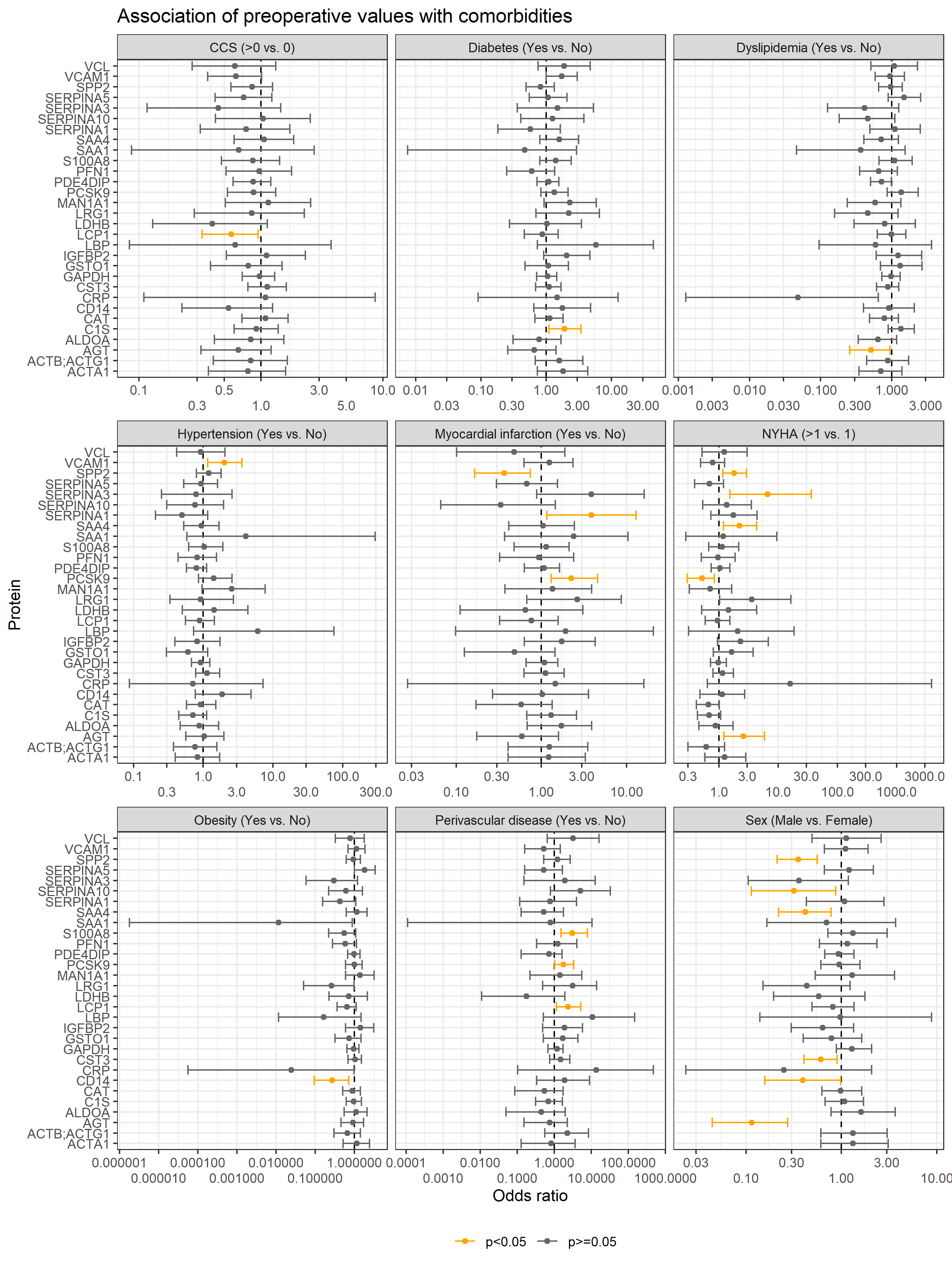
**

**Supplementary Figure 2.** Unadjusted associations of centered and scaled preoperative protein levels with continuous patient characteristics. Mean and 95%-confidence intervals of the regression coefficient in the univariable linear regression are shown.


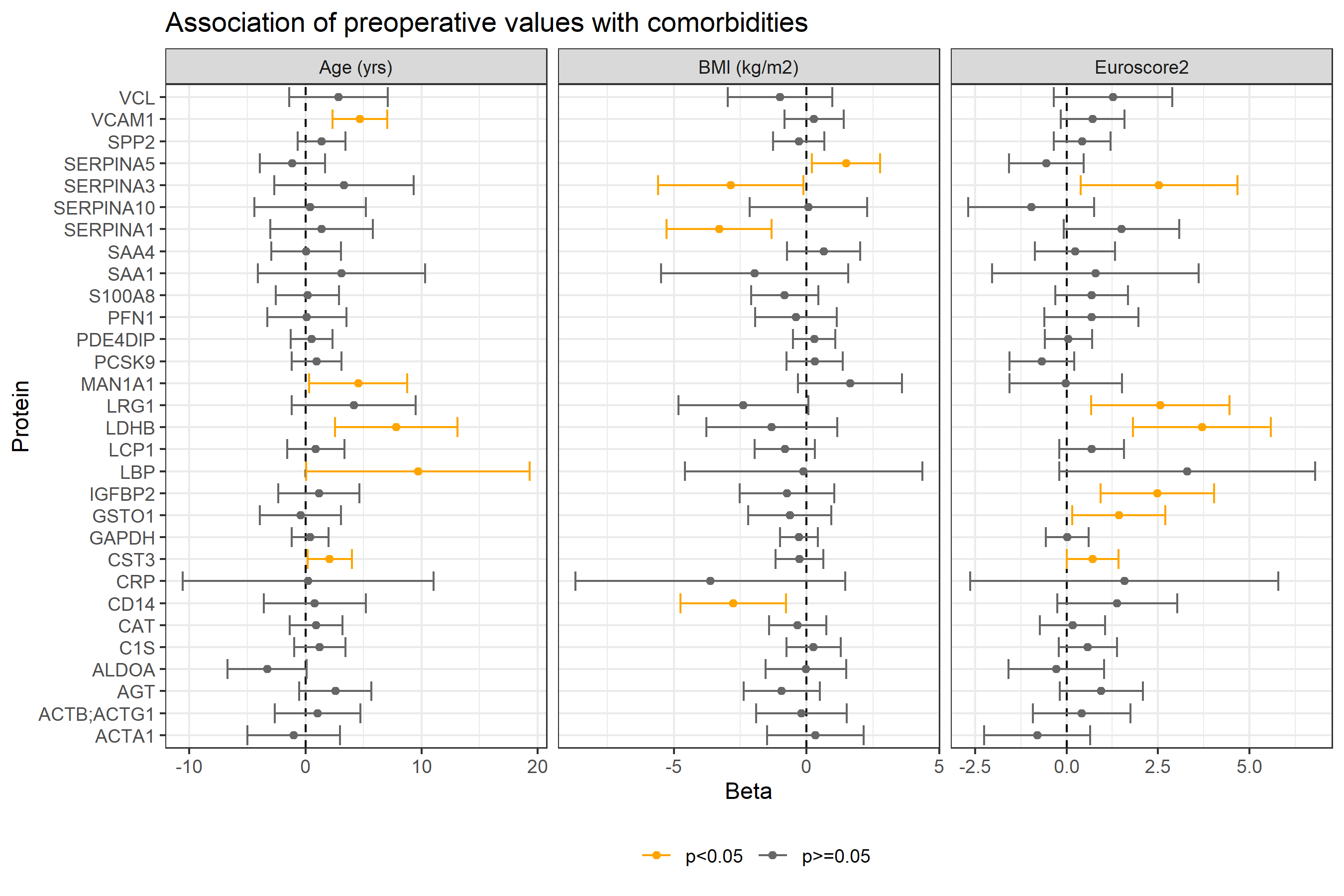


**Supplementary Figure 3.** Unadjusted associations of perioperative changes in protein levels with binary patient characteristics. Mean and 95%-confidence intervals of the odds ratios derived from univariable logistic regression are shown.


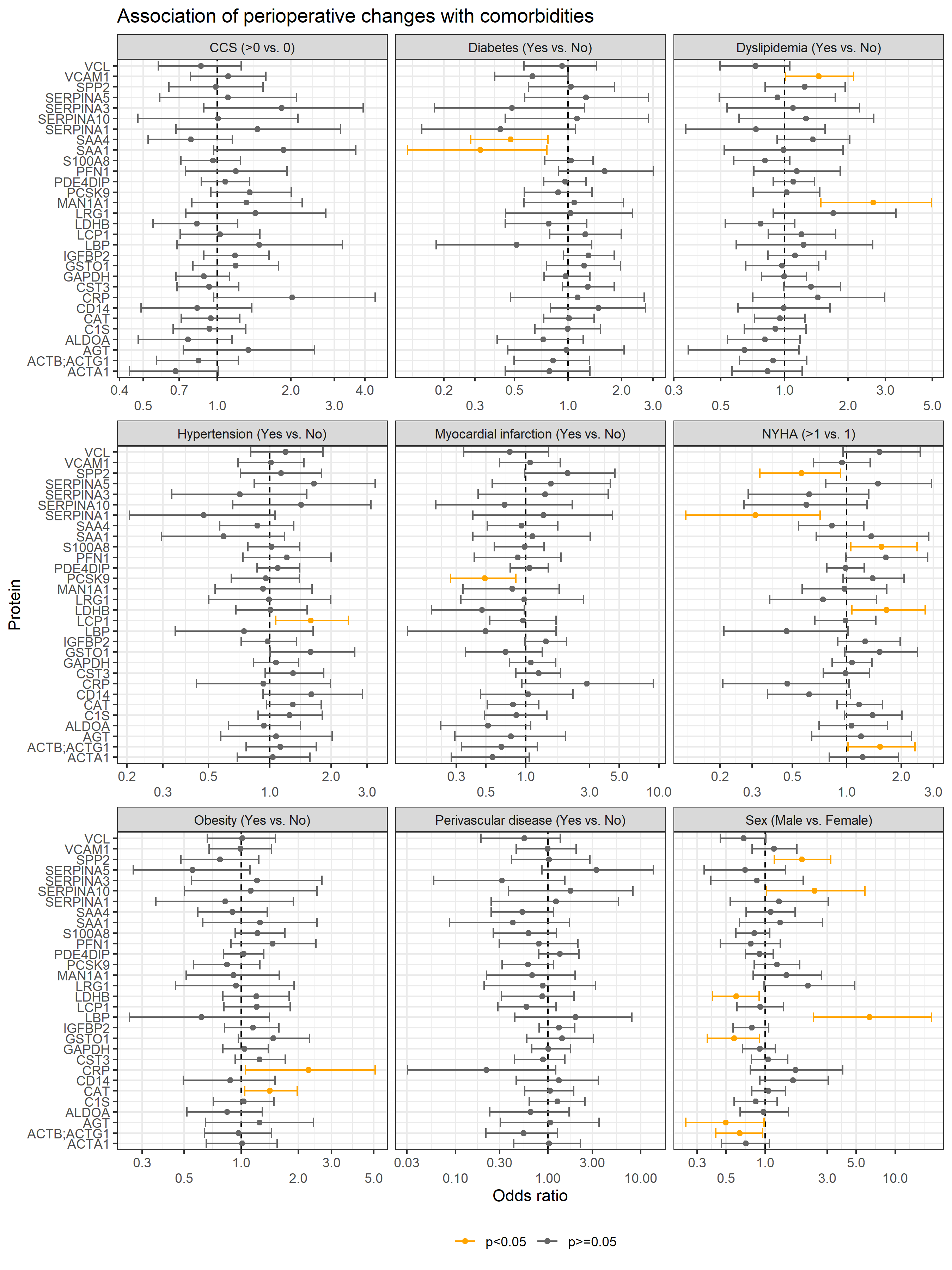


**Supplementary Figure 4.** Unadjusted associations of perioperative changes of protein levels with continuous patient characteristics. Mean and 95%-confidence intervals of the regression coefficient in the univariable linear regression are shown.


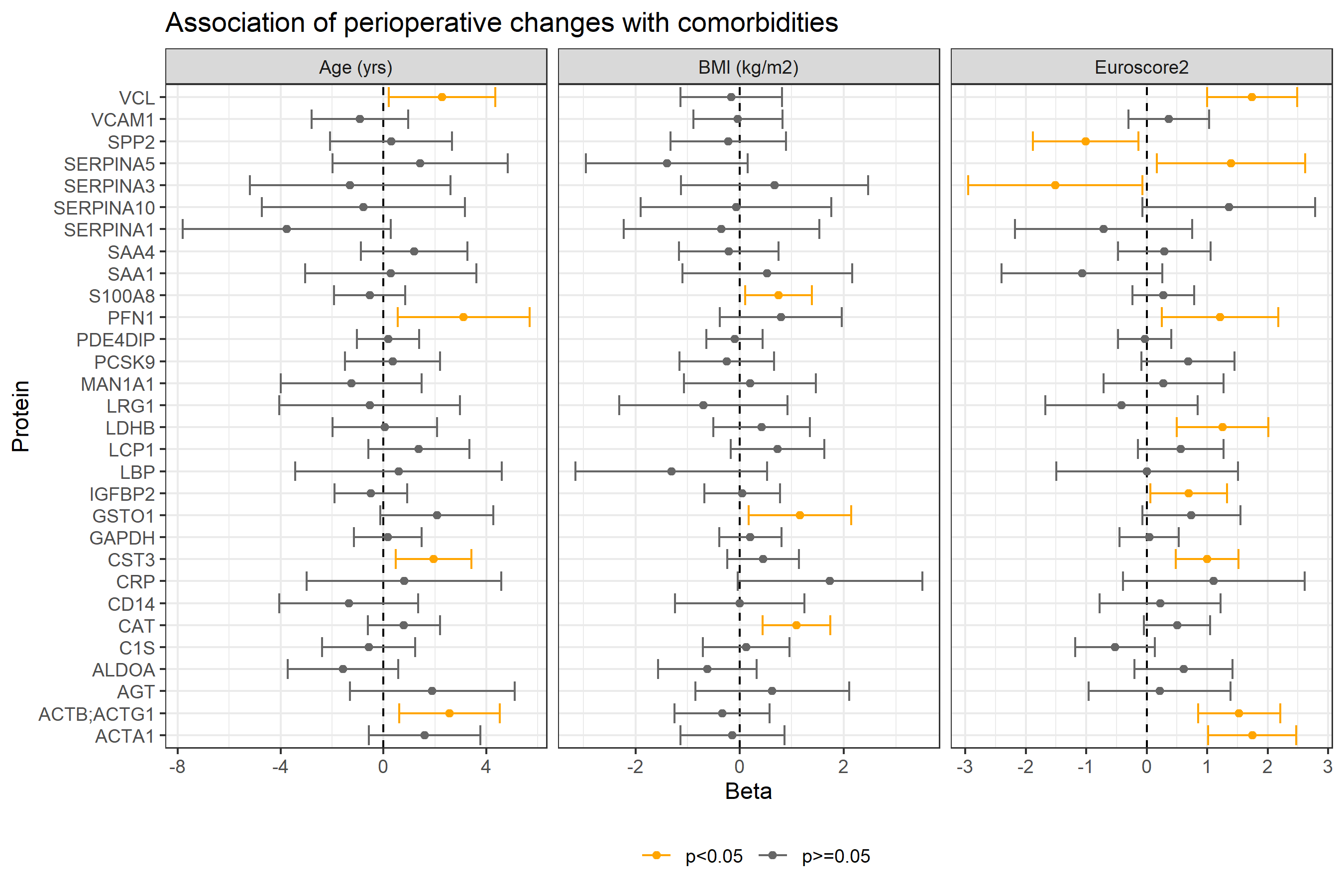

Supplement: Supplementary file 1 [file Datasheet1.docx]
